# Correlating semiconductor nanoparticle architecture and applicability for the controlled encoding of luminescent polymer microparticles

**DOI:** 10.1038/s41598-024-62591-1

**Published:** 2024-05-24

**Authors:** Lena Scholtz, J. Gerrit Eckert, Rebecca T. Graf, Alexandra Kunst, K. David Wegner, Nadja C. Bigall, Ute Resch-Genger

**Affiliations:** 1 Federal Institute for Materials Research and Testing (BAM), Division 1.2 Biophotonics, Richard-Willstätter-Str. 11, 12489 Berlin, Germany; 2 Institute for Chemistry and Biochemistry, Free University Berlin, Takustraße 3, 14195 Berlin, Germany; 3 Institute of Physical Chemistry and Electrochemistry, Leibniz University Hannover, Callinstraße 3A, 30167 Hannover, Germany; 4 Cluster of Excellence PhoenixD (Photonics, Optics, and Engineering − Innovation Across Disciplines), 30167 Hannover, Germany; 5 Laboratory of Nano- and Quantum Engineering, Leibniz University Hannover, Schneiderberg 39, 30167 Hanover, Germany; 6 Present Address: Department of Chemistry, University of Hamburg, Martin-Luther-King-Platz 6, 20146 Hamburg, Germany

**Keywords:** Chemistry, Materials science, Optics and photonics

## Abstract

Luminophore stained micro- and nanobeads made from organic polymers like polystyrene (PS) are broadly used in the life and material sciences as luminescent reporters, for bead-based assays, sensor arrays, printable barcodes, security inks, and the calibration of fluorescence microscopes and flow cytometers. Initially mostly prepared with organic dyes, meanwhile luminescent core/shell nanoparticles (NPs) like spherical semiconductor quantum dots (QDs) are increasingly employed for bead encoding. This is related to their narrower emission spectra, tuneability of emission color, broad wavelength excitability, and better photostability. However, correlations between particle architecture, morphology, and photoluminescence (PL) of the luminescent nanocrystals used for encoding and the optical properties of the NP-stained beads have been rarely explored. This encouraged us to perform a screening study on the incorporation of different types of luminescent core/shell semiconductor nanocrystals into polymer microparticles (PMPs) by a radical-induced polymerization reaction. Nanocrystals explored include CdSe/CdS QDs of varying CdS shell thickness, a CdSe/ZnS core/shell QD, CdSe/CdS quantum rods (QRs), and CdSe/CdS nanoplatelets (NPLs). Thereby, we focused on the applicability of these NPs for the polymerization synthesis approach used and quantified the preservation of the initial NP luminescence. The spectroscopic characterization of the resulting PMPs revealed the successful staining of the PMPs with luminescent CdSe/CdS QDs and CdSe/CdS NPLs. In contrast, usage of CdSe/CdS QRs and CdSe QDs with a ZnS shell did not yield luminescent PMPs. The results of this study provide new insights into structure–property relationships between NP stained PMPs and the initial luminescent NPs applied for staining and underline the importance of such studies for the performance optimization of NP-stained beads.

## Introduction

Luminescent polymer microparticles (PMPs) are frequently utilized in bioanalysis and medical diagnostics^1–4^. Typical applications are multimodal and multicolor labels, luminescence sensing of specific targets or biomolecular interactions^5,6^, drug carriers, and calibration beads^5–11^. Signal readout is typically performed optically using fluorescence spectroscopy, fluorescence microscopy or flow cytometry^12,13^. Combined with magnetic nanoparticles (NPs) like iron oxide NPs, such PMPs are also applied for immunoseparation^14–16^. For the preparation of such optically encoded beads, meanwhile different types of luminophores have been employed, including organic dyes, different semiconductor NPs, and lanthanide-based NPs^17–19^. Well suited candidates for the luminescence staining of PMPs, e.g., for multiplexing and encoding applications, are photostable quantum dots (QDs) and chemically inert, lanthanide-based upconversion NPs (UCNPs)^4,20–27^. The former reveal size-tunable absorption and emission bands, large molar absorption cross sections, narrow and symmetric emission bands, and high photoluminescence (PL) quantum yields (PLQY), while the latter exhibit a multicolor emission^4,20–27^. The colloidal nature, complex surface chemistry, and larger size of such luminescent NPs render PMP staining more challenging than the encoding with organic dyes. However, dye molecules are prone to photobleaching, concentration-dependent formation of non or barely emissive aggregates, and spectral crosstalk^22^. This introduces challenges for bead encoding with multiple fluorophores of varying emission color using a single excitation wavelength^28–30^. Due to this, an increasing number of reports on the staining of PMPs with luminescent NPs has been publsihed^24,27^. To tackle the challenges associated with the incorporation of NPs into PMPs, the NP surface can be coated with polymer compatible or even polymerizable ligands, which can improve the desired preservation of the initial luminescence properties of the NPs and prevent NP aggregation during bead synthesis^31–33^. Nevertheless, until now, most reports on NP-encoded PMPs were described the preparation of these beads and their applications^18,27,34,35^. There is still a significant lack of studies focusing on identifying best suited NP systems and morphologies, as well as on the reaction conditions for the improved preservation of the NP luminescence after PMP incorporation. Moreover, commonly only spherical NPs were employed for bead staining.

Despite the increasing usage of PMPs stained with spherical QDs made, e.g., from II/VI semiconductors, PMP encoding with semiconductor nanostructures of different size, shape, and chemical composition is still underexplored. This includes dot-in-rod systems of varying aspect ratios (length divided by width of the rod) with a core or dot located within an elongated nanorod-shaped surface passivation shell^36–38^, and 2D-nanomaterials like semiconductor-based nanoplatelets (NPLs)^39–41^. Favorable properties of the former, also referred to as semiconductor quantum rods (QRs), compared to classical QDs are their higher absorption cross sections and PLQY, especially for small aspect ratios^38^. NPLs have similarly advantageous size- and composition-dependent optical properties as QDs and QRs, yet exhibit more narrow emission bands and shorter luminescence lifetimes^39–41^. In addition, the spectral position of the NPL emission maxima can be tuned nearly independently of the lateral NPL dimensions^42–44^. Since NPLs are less chemically stable than the relatively robust QDs and QRs, their incorporation into polymer beads, which has not been reported so far, is expected to be even more challenging. This particularly applies to the conservation of their attractive luminescence features, e.g., their very narrow emission bands. Despite these possible challenges, NPLs as well as QRs can be considered as promising candidates for the staining of PMPs, e.g., as they can expand optical barcoding options using emission and luminescence lifetime read-out.

The tailored design of bright, NP-stained PMPs and the reproducible preparation of beads with controlled luminescence features can be eased by experimentally derived structure–property relationships. Particle architecture, composition, and surface chemistry can potentially influence the photoluminescence (PL) of the luminescent nanocrystals employed for encoding and its preservation, which can in turn result in changes of the optical properties of the NPs when incorporated into PMPs. This is particularly relevant for semiconductor QDs, QRs, and NPLs, where the chemical composition, thickness, and tightness of the surface passivation shell and the surface ligands largely control the optical properties of the NPs. This encouraged us to study the influence of the particle forming polymerization reaction on the incorporation and luminescence properties of representatively assessed CdSe-based nanostructures of different composition, shell thickness, and shape for the staining of polystyrene microparticles (PSMPs). Special emphasis was dedicated to the degree of PL preservation of the bead-incorporated NPs. NPs studied included CdSe QDs, made from the same CdSe core and surface passivated with CdS or ZnS shells of different thickness, CdSe/CdS QRs, and CdSe/CdS core/shell NPLs. To enable a comparison of the observed effects, the same polymerization protocol and identical reaction parameters were employed for all syntheses. This synthesis procedure relied on a protocol of the fabrication of PMPs stained with CdSe/CdS QDs, previously established by us^31,45^, which was adapted for this work. This includes the use of a polymer compatible ligand for NP surface coating prior to the polymerization reaction, which promotes PL preservation and prevents NP leakage from the NP-stained beads^31^. As measures for polymerization-induced changes in NP size and/or size distribution and quality of the core surface passivation, we utilized the spectral position and width of the PL band, the PLQY as well as the PL decay kinetics. Electron microscopy was employed to determine the size and size distribution of the NP-stained PSMPs and the spatial distribution of the NPs within the beads. Based upon the results of this screening study, a correlation between the degree of PL preservation during the polymerization and NP architecture could be derived. NP properties were identified that are particularly relevant for retaining a high PL of the semiconductor nanostructures within the PSMPs, such as NP surface chemistry and shell thickness.

## Materials and methods

The experimental procedures employed for the synthesis of the CdSe/CdS QDs and CdSe/CdS NPLs, polyethylene glycol-*block*-poly(ε-caprolactone) as well as the synthesis of QD stained PSMPs and the performance of the fluorescence and integrating sphere spectroscopy and atomic absorption spectrometry measurements were partly employed and reported by us before^31,39,45^.

### Materials

Styrene (≥ 99.0%), tin(II) 2-ethylhexanoate (92.5–100%), ε-caprolactone (97%), poly(ethylene glycol) (PEG, M_W_ 2,500), divinylbenzene (DVB, 80%), azobisisobutyronitrile (AIBN, 98%), trioctylphosphine oxide (TOPO, 99%), 1-octadecene (ODE, 90%), isopropanol (≥ 99.8%), oleylamine (OLA, for QD synthesis, 70–80%), 1-octanethiol (98.5%), Na(myristate) (≥ 99%), *n*-hexane (≥ 99%), methanol (≥ 99.8%), ethanol (≥ 99.8%) and toluene (≥ 99.7%) were purchased from Sigma Aldrich Co. Cadmium oxide (CdO, 99.998%), Se powder (200 mesh, 99.999%), Cd(NO_3_)_2_·4 H_2_O (99.999%), polyvinylpyrrolidone (PVP, M_W_ 40,000) and oleic acid (OA, 90%) were obtained from Alfa Aesar. Ethanol, *n*-heptane and toluene (all spectr. grade) as well as *n*-hexane (≥ 99%) were purchased from Merck KGaA. Dichloromethane (HPLC grade) and ethanol (abs., 99.9%) were obtained from Chemsolute, tri-*n*-octylphosphine (TOP, 99.7%), Cd(acetate)_2_·2 H_2_O (98%) and deuterated chloroform (99.8 atom%) as well as the CdSe/CdS QRs (CANdot quantum rods, 5 mg/mL, product nr. AB391053) from abcr GmbH. Benzyldimethyloctadecylammonium chloride (OBDAC, 98.9%) was obtained from HPC Standards GmbH, *n*-octadecylphosphonic acid (ODPA, > 99%) from PCI Synthesis, and oleylamine (OLA, for NPL synthesis, 80–90%) from Acros Organics (now Thermo Scientific Chemicals). All chemicals were employed without further purification, all solvents used for optical measurements were of spectroscopic grade. All aqueous solutions were prepared with deionized water (0.055 μS∙m^-1^; Milli-Q water, Millipore).

### Synthesis of CdSe/CdS and CdSe/ZnS-core/shell QDs

CdSe/CdS-core/shell QDs with shell thicknesses of about 3, 5, and 10 monolayers (ML) surface stabilized with oleic acid and oleylamine were prepared from the same CdSe core particles according to a modified synthesis adapted from Carbone et al. Nightingale et al. and Chen et al.^20,21^ which was previously partly described by us^31,45^. The same CdSe cores were also employed for the synthesis of CdSe/ZnS QDs with a ZnS shell thickness of approximately 3 ML. The shell growth was carried out according to a self-developed procedure by J. G. Eckert, and the Zn precursor synthesis according to Boercker et al.^47^, respectively. The NP syntheses are described in detail in the Supporting Information (SI).

### Synthesis of CdSe/CdS-core/shell NPLs

CdSe/CdS-core/shell-NPLs with a shell thickness of 4.5 ML, surface stabilized with oleic acid and oleylamine, were synthesized according to a procedure adapted from Tessier et al. Abécassis et al. Miethe et al. and Rossinelli et al.^48–51^ The NPL synthesis is detailed in the SI.

### Synthesis of CdSe/CdS-dot-in-rod QRs

The CdSe/CdS-dot-in-rod QRs surface stabilized with octadecylphosphonic and hexylphosphonic acid ligands were purchased from abcr GmbH (CANdot quantum rods, product nr. AB391053).

### Synthesis of polyethylene glycol-*block*-poly(ε-caprolactone)

The synthesis of the *block*-copolymer polyethylene glycol-*block*-poly(ε-caprolactone) (PEG-*b*-PCL) was performed following a previously reported procedure^31^ adapted from Meier et al.^52^ which is detailed in the SI. The chemical identity of the synthesized PEG-*b*-PCL was confirmed by solution ^1^H-NMR spectroscopy (see SI, Fig. S1).

### Synthesis of NP-stained polystyrene microparticles (PSMPs)

The synthesis of the NP-stained PSMPs was performed according to a procedure recently reported by us with minor modifications^31,45^. First, all luminescent NPs explored were coated with OBDAC to ensure a better compatibility with the polymer matrix of the PSMPs to be fabricated. The addition of OBDAC, in combination with the subsequently performed crosslinking of the polymer matrix, had proved to be very beneficial for retaining the initial PL of NPs and the prevention of NP leakage in previous works^31,45^, and was hence also utilized in this study. Therefore, a spatula tip of OBDAC (about 2 mg) was added to 300 µL of each NP solution (all 0.96 mg Cd/mL). Ethanol was added until a volume of 1 mL was reached, and the mixture was placed on a shaker for 5 min. The precipitated NPs were then centrifuged at 6000 *rcf* for 5 min, the supernatant was discarded, the NPs were redispersed in fresh ethanol, centrifuged again using the same parameters as before, and the supernatant discarded. Finally, the resulting OBDAC-coated NPs were redispersed in 1.1 mL styrene, sealed, and stored in the refrigerator until use in the polymerization reaction.

For the synthesis of the NP-stained PSMPs, 45.75 mg PEG-*b*-PCL were added to 504 µL toluene, and the mixture was placed on a shaker for 30 min. In the meantime, 400 mg PVP (M_W_ 58,000) were dissolved in 45 mL of ethanol. A 100 mL two-neck flask was purged with argon for 5–10 min, and both solutions were combined in the flask under argon flow. The flask was sealed, equipped with an argon balloon, heated to 80 °C in an aluminum heating block, and the mixture was stirred for 30 min at 100 rpm. In parallel, 1 mL of the OBDAC-coated NP dispersion were combined with 4 mL styrene and 50 µL DVB, and the mixture was briefly sonicated. Additionally, 180 mg AIBN were dissolved in 5 mL ethanol. The NP dispersion was added first to the PEG-*b*-PCL containing reaction mixture, followed by addition of the AIBN solution. The polymerization reaction was allowed to proceed for 4 h at 80 °C using a stirring speed of 100 rpm. After cooling, the polymerization mixture to RT, the formed NP-stained PSMPs were centrifuged for 2 min at 2000 *rcf*, and the supernatant was discarded. The PSMPs were then redispersed in ethanol, centrifuged under the same conditions as employed before, the supernatant was discarded, and the beads were redispersed in 45 mL ethanol. Before further application/characterization of this stock solution of NP-stained PSMPs, a defined amount of PSMPs was taken off the stock solution, centrifuged for 2 min at 1600 *rcf*, the supernatant was discarded, and the particles were redispersed in ethanol. This procedure was repeated two more times.

### Atomic absorption spectroscopy (AAS)

AAS measurements were performed with an AA140 instrument from Varian Inc. with an air/acetylene flame atomizer to determine the cadmium ion (Cd(II)) concentration in the NP dispersions. Samples of the NP dispersions were prepared by dissolution of the particles with *aqua regia*. Six standard solutions with different Cd(II) concentration (0–2.5 ppm) were used to obtain a calibration curve for the subsequent quantification of the Cd(II) concentration.

### Scanning electron microscopy (SEM)

The NP-stained PSMP samples were measured with a Philips XL30 ESEM using an acceleration voltage of 25 kV. The samples were prepared by drop-casting directly onto aluminum holders from diluted, ethanolic dispersions. The mean particle sizes and the corresponding size distribution of all PSMP samples were determined using the software ImageJ (Version: 1.52e, https://imagej.nih.gov/ij/).

### Transmission electron microscopy (TEM)

The TEM images of the CdSe/CdS QDs with a 5 ML thick surface passivation CdS shell and the CdSe/CdS-dot-rods, drop-casted onto carbon-coated copper grids (PELCO by Ted Pella, Inc., 400 mesh), were measured with a Thermo Fisher Scientific Talos F200S TEM at 200 kV. TEM measurements of all other NP dispersions were conducted with a JEOL JEM-2100F-UHR equipped with a field emission gun, operated at 200 kV. The NP samples were prepared on carbon-coated copper grids (Quantifoil, 400 mesh) via drop casting. The mean particle sizes and the size distributions of all NPs were determined with ImageJ as described above for the SEM samples.

### High-angle annular dark-field scanning transmission electron microscopy (HAADF-STEM)

For the HAADF-STEM measurements of the NP-stained PSMPs, the samples were drop-casted from diluted PSMP dispersions in ethanol onto carbon-coated copper grids (PELCO by Ted Pella, Inc., 400 mesh). For the NPL-stained PSMPs, lacey grids with otherwise the same specifications were used. Imaging was performed with a ThermoFisher Scientific Talos F200S TEM at 200 kV.

### Fluorescence spectroscopy

The emission spectra of the NPs in hexane and the emission spectra of the NP-stained PSMPs in ethanol were recorded with a calibrated FSP920 fluorescence spectrometer from Edinburgh Instruments Ltd. at RT ((25 ± 2) °C) using (10 × 10) mm quartz glass cuvettes (Hellma GmbH). Excitation was always at 350 nm.

Measurements of the PL decay curves of these samples, the fits of which providing the respective fluorescence lifetimes (FLTs), as described in the SI (see Eqs. SE1 and SE2) were performed with a calibrated FLS920 fluorescence spectrometer from Edinburgh Instruments Ltd. in (10 × 10) mm quartz glass cuvettes (Hellma GmbH) at RT. The samples were excited with a 375 nm EPL picosecond pulsed diode laser from Edinburgh Instruments Ltd.

### Integrating sphere spectroscopy

The PLQY values of the NP-stained PSMPs in ethanol and the semiconductor NP dispersion in toluene were determined with a stand-alone Quantaurus integrating sphere setup (Hamamatsu Photonics K.K.). The absolute PLQY measurements were performed in (10 × 10) mm long-neck quartz glass cuvettes (Hamamatsu Photonics K. K.) at RT using an excitation wavelength of 350 nm. As a blank for the transparent NP dispersions, the respective solvent was used. For the scattering NP-stained PSMPs, a blank containing a dispersion of unstained PSMPs of matching size and bead concentration was employed.

## Results and discussion

**Figure 1:**
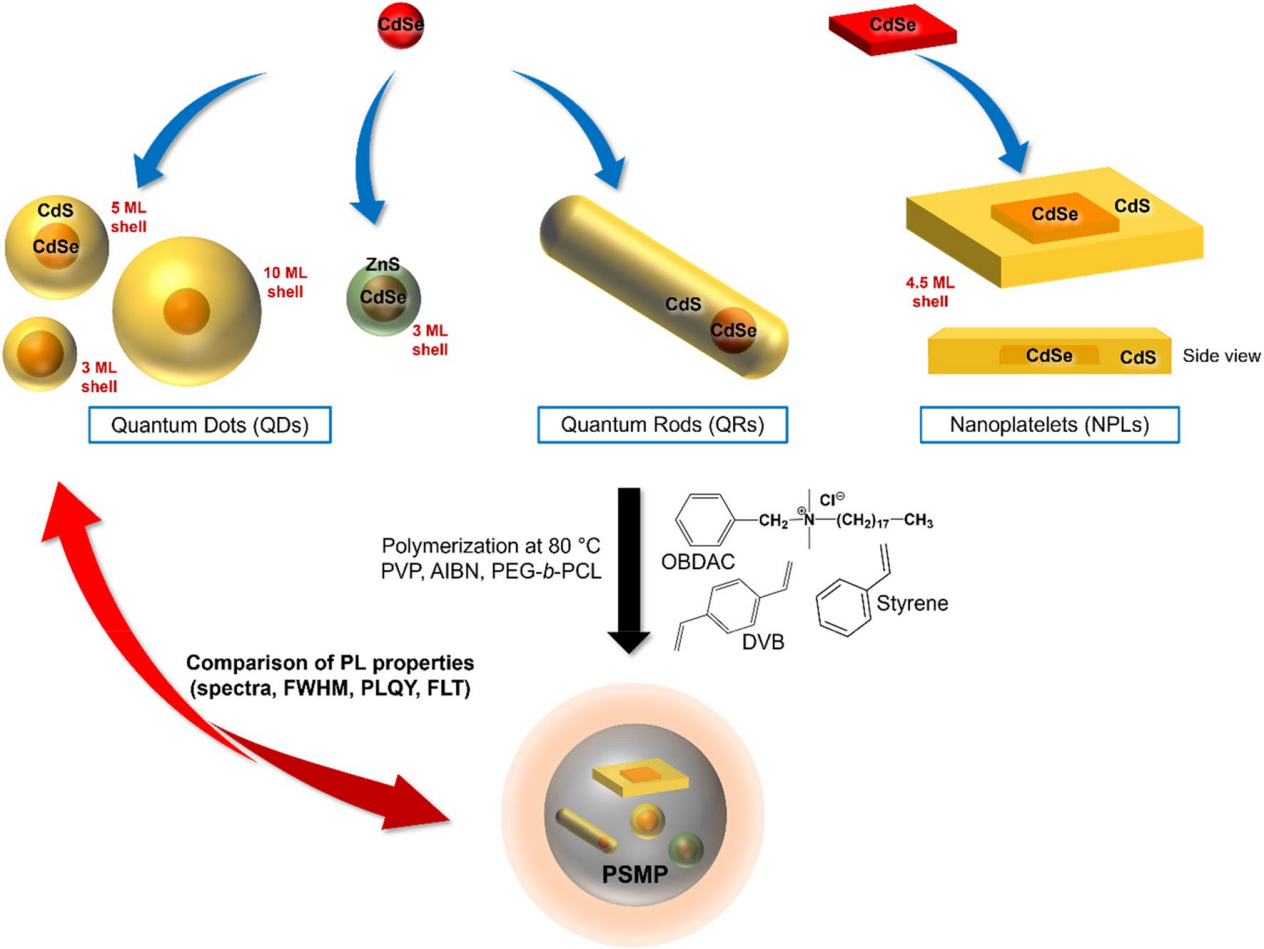
Schematic representation of the different types of used semiconductor NPs and an overview of the synthesis of NP-stained PSMPs. Employed NPs include (i) spherical core/shell CdSe/CdS QDs of varying thickness of the surface passivation shell and (ii) spherical core/shell CdSe/ZnS QDs, as well as (iii) CdSe/CdS-dot-in-rod QRs, and (iv) CdSe/CdS-core/shell NPLs. All NPs bear oleic acid (OA) and oleylamine (OLA) surface ligands, except for the QRs that are surface stabilized with octadecylphosphonic acid and hexylphosphonic acid. All QDs and NPLs were supplied by the Bigall group at LUH, the QRs were purchased from abcr GmbH (CANdot quantum rods, product nr. AB391053). The polymer matrix is formed from styrene and divinylbenzene (DVB), AIBN is used as an initiator, and PVP as a surfactant. The amphiphilic copolymer PEG-*b*-PCL is added to realize a narrower size distribution, and the additional ligand OBDAC provides a better NP compatibility with the polymer matrix. PL: photoluminescence, FWHM: full width at half maximum of the PL band, PLQY: photoluminescence quantum yield, FLTs: fluorescence lifetimes, determined from the measured and fitted photoluminescence (PL) decay kinetics (see SI, Eqs. SE1 and SE2).

The CdSe-based nanostructures representatively employed for PSMP staining in this screening study aiming for establishing a correlation between the architecture of core/shell semiconductor nanomaterials and the application-relevant physicochemical properties of the NP-stained PSMPs are displayed in Fig. 1. As a prerequisite for the desired comparison of NP-specific effects and the identification of optimum NP architectures for PSMP encoding, we always used the same polymerization protocol and identical reaction conditions. This protocol was previously developed by us for CdSe/CdS QDs^31,45^ and adapted for this work.

The synthesis of the NP-stained PSMPs is also summarized in Fig. 1. The luminescence properties of the representatively chosen CdSe-based QDs, QRs, and NPLs and the respective polymerization- and environment-induced changes in the PL properties of these NPs were derived from measurements of the emission spectra, PLQY, and fluorescence decay kinetics. Also, other application-relevant bead parameters were assessed, such as the size and size distribution of the NP-stained PSMPs, and the NP distribution within the PSMPs. The determination of the NP dispersion concentrations was performed by AAS and the size and size distribution of the different NP systems was derived from TEM image analysis. The size and size distribution of the NP-stained PSMPs was obtained from SEM image analysis and the NP spatial distribution within the beads from HAADF-STEM measurements.

### Physicochemical and optical properties of the luminescent semiconductor NPs

**Figure 2:**
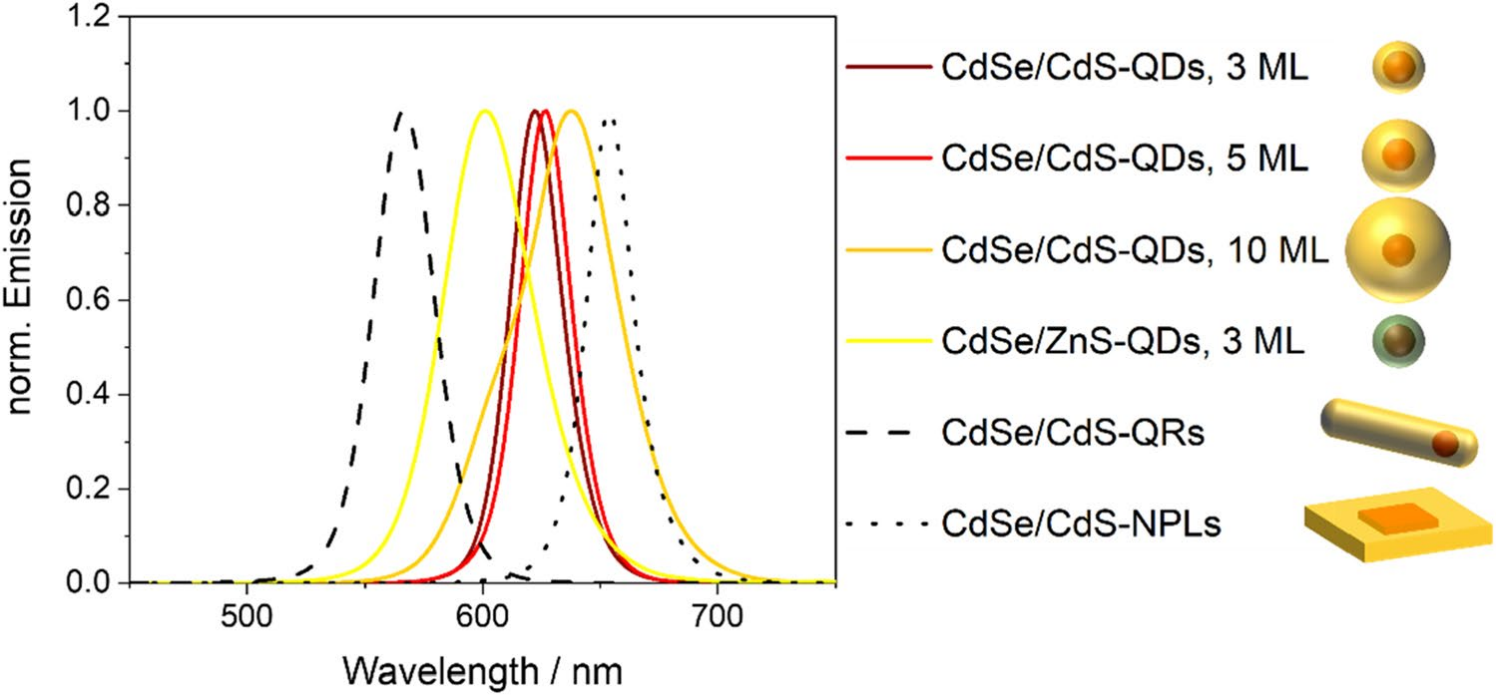
Emission spectra (λ_exc_ = 350 nm) and a schematic representation of the different luminescent semiconductor nanostructures employed in this work, that were all dispersed in hexane. The absorption spectra of the NPs are provided in the SI (see Fig. S2). All QDs were prepared from the same core particles, except for the CdSe/CdS QDs with a 5 ML CdS shell. Here, slightly smaller CdSe cores with a size of 3.6 nm compared to 3.9 nm were employed. All QDs and NPLs were supplied by the Bigall group at LUH, the QRs were purchased from abcr GmbH (CANdot quantum rods, product nr. AB391053).

Table 1 and Fig. 2 summarize the relevant physicochemical properties of the QDs, QRs, and NPLs used in this study. These NPs reveal narrow and symmetric PL bands in the wavelength region of about 530 nm to 700 nm except for the CdSe/CdS QDs with a 10 monolayer (ML) CdS shell, which show a broadened PL band. This indicates a slightly broader size distribution caused by the one-step, extended shelling procedure required to achieve a 10 ML CdS shell. The influence of the shell material employed for the surface passivation of the CdSe core (CdS or ZnS) is reflected by the spectral position of the emission band, PLQY value, and the PL decay kinetics. These were fitted with the equations SE1 and SE2 given in the SI, yielding the average fluorescence lifetimes (FLTs). For the CdSe/CdS core/shell QDs, the increase in shell thickness leads to an increase in QD size and a bathochromic shift of the emission band as well as a decrease in PLQY and an increase in FLT as reported in the literature^53,54^. The latter is ascribed to the different delocalization of the wave functions of electron and hole into the shell^55^. In a quasi-type II semiconductor heterostructure like CdSe/CdS QDs, the electron with its lower effective mass can be significantly delocalized into the shell, while the heavier hole is located in the core. This implies that with increasing shell thickness, the electron is delocalized over a larger volume, which enhances the time required for the radiative recombination of the exciton^54^. The ZnS surface shell introduces a hypsochromic shift of the emission band compared to the CdSe/CdS QDs. Moreover, a comparison of the CdSe QDs with a 3 ML CdS and 3 ML ZnS shell reveals significantly longer lifetimes for the CdSe/ZnS QDs and a significantly lower PLQY (see also Table 1). As follows from Table 1, the optical properties of semiconductor nanostructures are not only largely dependent on material composition, but also on particle shape as reported extensively in the literature^56,57^. The emission maximum of the QRs is directly linked to the aspect ratio (l/w) of the QRs. This equals 2.9 for the QRs used in this work. In the case of the NPLs, the shell growth procedure determines the type of the semiconductor heterostructure of the resulting NPL. The CdS shell shields the CdSe core in three dimensions for the CdSe/CdS core/shell QDs, yielding a quasi-type II semiconductor heterostructure with a core/shell architecture^51^. This protects the emissive core from environmental effects, and enhances the core fluorescence.

**Table 1:** Overview of the physico-chemical properties of the different semiconductor NPs assessed with a CdSe core including their application-relevant luminescence properties (λ_em_: emission maximum; PL QY: photoluminescence quantum yield).

Shape	Shell & thickness	Particle size/nm	λ_em_/nm	QY/%	FLT/ns
Spherical	CdS, 3 ML	7.9 ± 0.9	622	78	12 & 32
	CdS, 5 ML	7.9 ± 0.7	627	67	20 & 36
	CdS, 10 ML	10.3 ± 1.2	638	59	73 & 184
	ZnS, 1 ML	5.1 ± 0.6	601	45	20 & 60
Dot-in-rod	CdS	4.0 ± 0.5 11.5 ± 2.2	566	86	5 & 16 & 65
2D Platelet	CdS	16.6 ± 1.8 17.2 ± 2.2	654	34	2 & 10 & 44

**Figure 3:**
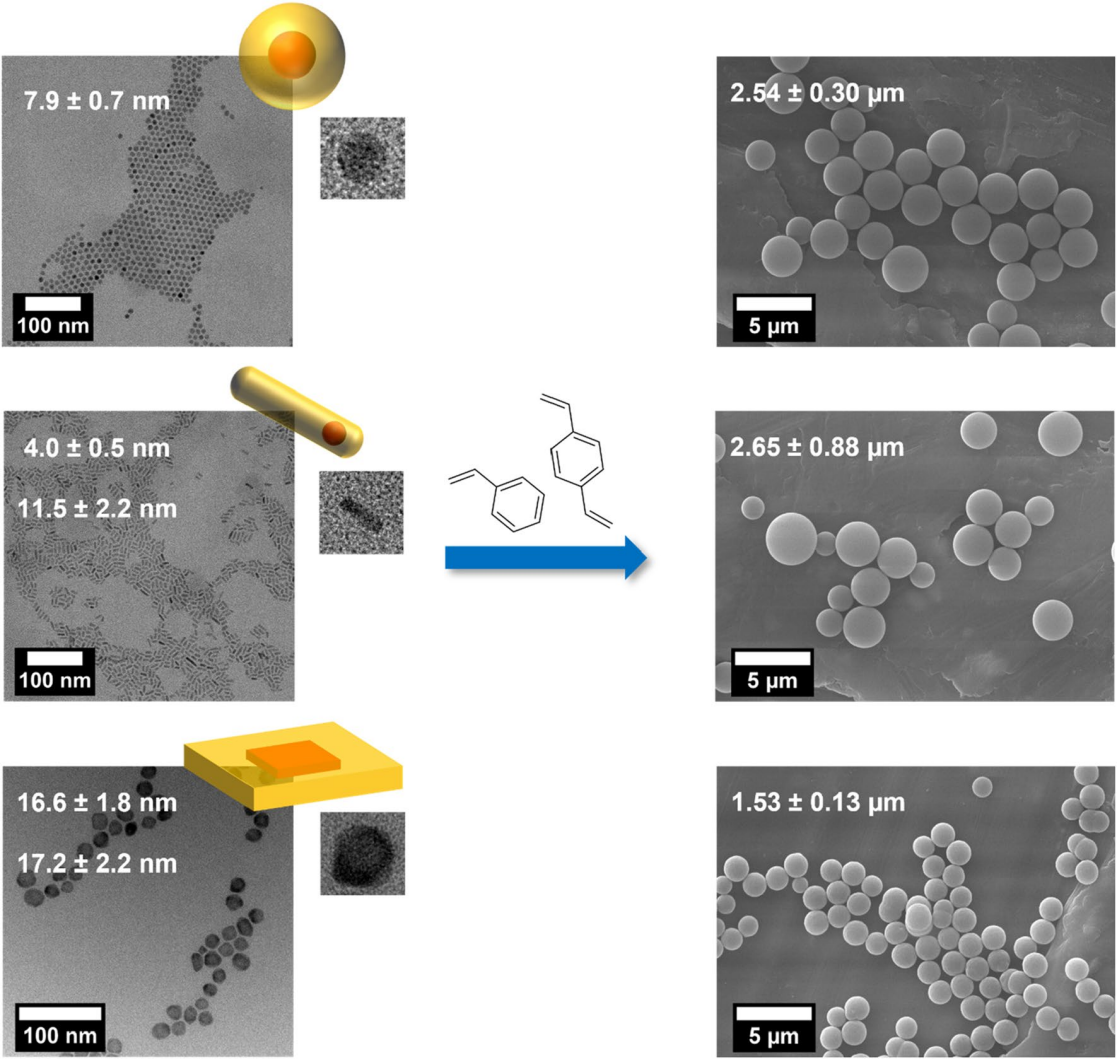
TEM images (left) of representative CdSe/CdS QDs (5 ML CdS shell), CdSe/CdS QRs, and CdSe/CdS NPLs, including the average particle sizes; and SEM images (right) of the resulting, NP-stained PSMPs. The histograms reflecting the size distribution of the NPs and PSMPs and the TEM/SEM images of the other NPs and the NP-stained PSMPs studied are provided in the SI (see Figs. S3–S5). Individual, close-up images of the different NPs are provided as insets with the following image sizes: 15 × 15 nm (QDs), 17 × 17 nm (QRs), and 23 × 23 nm (NPLs).

The different spherical CdSe-based QDs were prepared from the same CdSe core particle batch, except for the CdSe/CdS QDs with a 5 ML thick CdS shell that was made from a different CdSe core particle batch with closely matching size. The similar size of the 3 ML and 5 ML QDs is attributed to the slightly smaller CdSe core utilized for the synthesis of the latter core/shell CdSe/CdS QDs. The size distributions of the NPs and the multiexponential PL decay curves, from which the FLTs were obtained, are provided in the SI (see Fig. S2 and Eqs. SE1 and SE2). The emission spectra and TEM images of the NPs are displayed in Figs. 2 and 3.

### Structure-analytical characterization of the NP-stained PSMPs

Figure 3 displays representative TEM and SEM images of the luminescent NPs (left panels) and the corresponding NP-stained PSMPs (right panels), shown here exemplarily for a CdSe/CdS QD sample (5 ML CdS shell), the QRs, and the NPLs.

As follows from the SEM images, all NP-stained PSMPs are spherical and have a very smooth surface, except for the NPL-stained PSMPs, which have a very slightly rough/textured surface structure. The size of the QD- and QR-stained PSMPs ranges between 2–3 µm, while the size of the NPL-stained particles is significantly smaller with a value of about 1.5 µm. The PSMP size distribution is relatively narrow for the NPL- and QD-stained PSMPs (8.5% for NPLs and 11.1/11.8/10.4% variation for 3/5/10 ML QDs, respectively), except for the PSMPs stained with the CdSe/ZnS QDs (33.1% variation). It is also significantly broadened for the QR-stained PSMPs (33.2% variation).

Apparently, the type of semiconductor nanostructure added to the polymerization reaction mixture can influence the size and size distribution of the resulting NP-stained PSMPs, and slightly even their surface morphology. Especially the anisotropic NPs, i.e., the QRs and particularly the NPLs, seem to have a significant influence on the size of the resulting NP-stained PSMPs. In a previous work, we showed that addition of CdSe/CdS QDs with a 5 ML thick CdS shell does not have a significant influence on the size of the PSMPs compared to unstained particles prepared under identical conditions of the polymerization reaction^31^. The observed influence of semiconductor NP shape as well as NP architecture, i.e., the inorganic surface passivation shell and the stabilizing organic ligand shell, on the size of the resulting NP-stained PSMPs suggests that the encoding NPs can influence the polymerization process leading to PSMP formation.

### Spatial distribution of the semiconductor NPs within the PSMPs

The applicability of the PSMPs stained with luminophores can be also affected by the spatial distribution of the luminescent nanostructures utilized for bead encoding. Information on the homogeneity of PSMP staining and the localization of the NPs within the PSMPs is especially relevant here as, e.g., NPs incorporated close to the PSMP surface could possibly affect further surface functionalization steps or favor NP leakage. In addition, the NP spatial distribution within the NP-stained PSMPs can provide a hint at which step of the bead forming polymerization reaction the NPs are incorporated. This can contribute to a better understanding of the initial steps and propagation of the dispersion polymerization reaction. This information can be important for the optimization of the NP particle architecture and the polymerization reaction conditions, which are required to further improve the luminescence properties of the PSMP-encapsulated NPs, particularly the preservation of the luminescence features of the encoding NPs.

**Figure 4:**
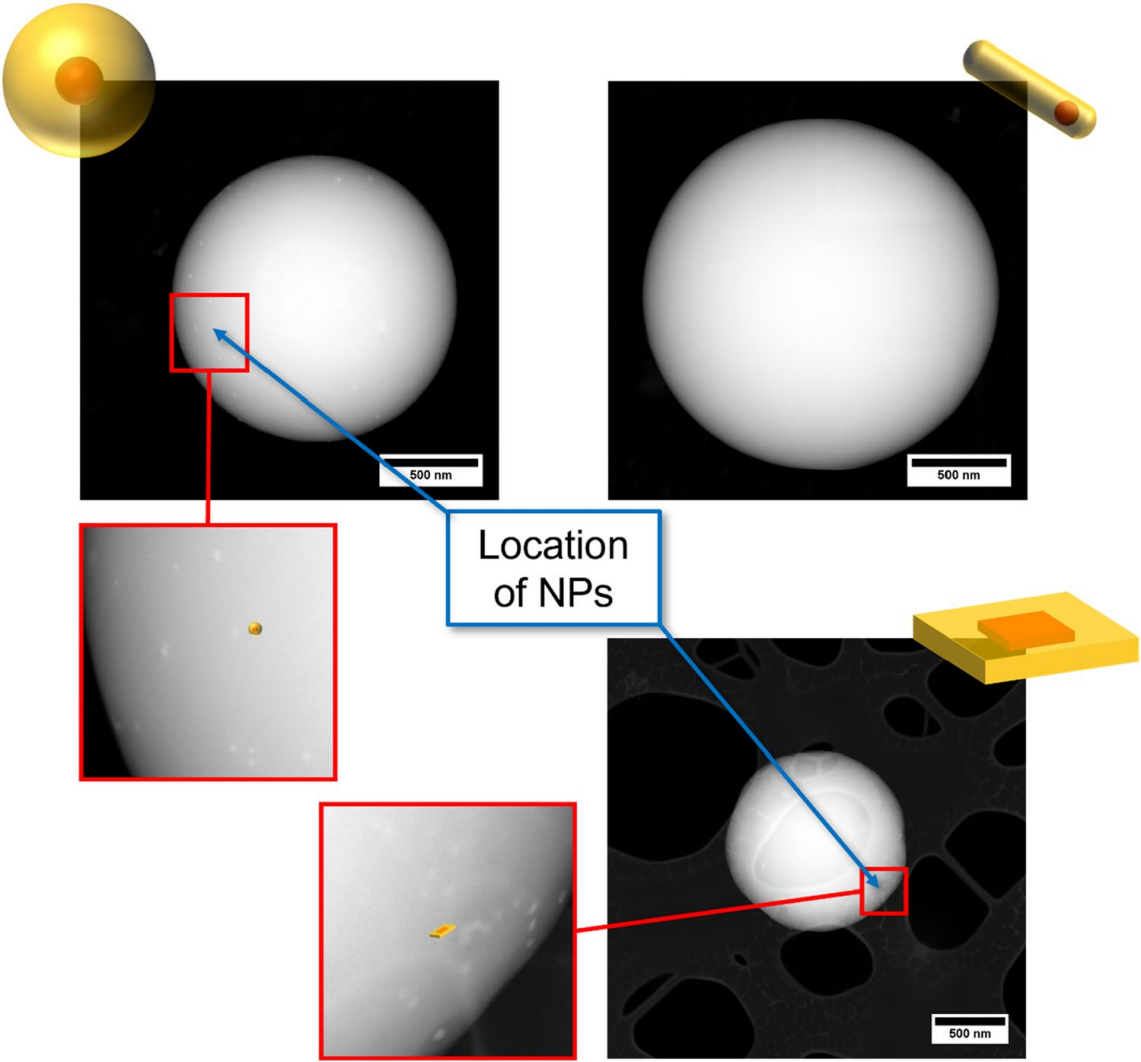
STEM images of NP-stained PSMPs, representatively shown for CdSe/CdS QDs with a 10 ML CdS surface passivation shell (top, left), CdSe/CdS QRs (top, right), and CdSe/CdS NPLs (bottom, right). The location of the semiconductor NPs is marked and displayed in more detail for smaller regions of the polymer particles. In the case of the QR-stained PSMPs, no QRs could be detected within the PSMPs. Comparative STEM images of COOH-functionalized PSMPs stained with CdSe/CdS QDs bearing a 10 ML CdS shell and plain PSMPs stained with CdSe/CdS QDs with a 5 ML CdS shell are displayed in the SI (see Fig. S8). The patchy areas in the background of the NPL-stained PSMPs correspond to a different, lacey TEM grid that was employed for the measurement in this case.

We determined the location of the different types of semiconductor nanostructures by recording HAADF-STEM images of the NP-stained PSMPs that are highlighted for selected samples in Fig. 4. HAADF-STEM is a dark field mode of TEM imaging. In HAADF-STEM measurements, not the primary, transmitted electron beam (selected for bright field imaging) is detected but the electrons diffracted by the sample. This is done with an annular detector located around the primary beam above the sample in a high angle. Due to the detection of scattered electrons, HAADF-STEM is much more sensitive to changes in the atomic number of the sample atoms compared to the bright field mode and can detect small, crystalline structures in PSMPs. As the atomic numbers of the polymer matrix components (mostly 1 and 6 for H and C) and the inorganic NP components (48 for Cd, 30 for Zn, 34 for Se and 16 for S) largely differ, the contrast in HAADF-STEM measurements is sufficient and allows to distinguish both materials. In conventional bright field imaging, due to the high intensity of the primary electron beam, the signals from small crystalline structures are often covered and can thus become indistinguishable from the surrounding material. In dark field TEM methods such as HAADF-STEM, a higher atomic number corresponds to scattered electrons with higher energy resulting in brighter areas in the displayed image. Therefore, the NPs show up as small, bright dots or sheets in the polymer matrix of the PSMPs. As illustrated in Fig. 4, the CdSe/CdS QDs and the CdSe/CdS NPLs are detectable within the PSMPs. Both NPs are preferentially located in the surface regions of the respective NP-stained PSMPs. These QDs and NPLs as well as the CdSe/ZnS QDs (which have not been further analyzed by STEM because of PL quenching) are stabilized with a mixture of oleic acid and oleylamine ligands. A near surface location of oleic acid- and oleylamine-capped CdSe/CdS QDs in PSMPs was observed by us in an earlier study on the QD staining pf PSMPs surface functionalized with COOH surface groups (utilizing acrylic acid)^45^. For these COOH-surface functionalized PSMPs, we ascribed the near surface location of these QDs to an interaction of the QDs with the carboxylic acid functionalities on the bead surface (see also STEM image in Fig. S8 in the SI). The observation of a similar spatial distribution for the oleic acid- and oleylamine-capped CdSe/CdS QDs for the NP-encoded PSMPs prepared in this study, where no COOH surface groups are present, suggests that in our study, the NPs do not act as nucleation points from which the formation and growth of the PSMPs starts. Apparently, the oleic acid and oleylamine capped QDs and NPLs are for our polymerization procedure more likely incorporated at a later stage of the polymerization reaction. Nevertheless, they can seemingly still influence the dynamics of the polymerization reaction. The observation that the differently shaped CdSe/CdS NPLs, that bear the same surface capping ligands as the spherical CdSe/CdS QDs, are also preferentially located in the surface regions of the polymer beads suggests a considerable influence of NP surface chemistry on the incorporation of the NPs into the PSMPs formed by the applied dispersion polymerization protocol. Nevertheless, preliminary leaking studies confirmed that the NPs are tightly incorporated into the PSMPs or PSMP surface region. In addition, while, e.g., the luminescence of oleic acid- and oleylamine-capped CdSe/CdS QDs in ethanol is quickly quenched, storage of the NP-encoded beads in ethanol leads only to a slight time-dependent deterioration of the NP luminescence. The spatial location of the NPs in the surface region of the PSMPs could be possibly beneficial for applications with Förster resonance energy transfer (FRET) processes if the distance between these NPs and a FRET donor or acceptor is < 10 nm.

In contrast, the STEM images of the QR-stained PSMPs do not provide a hint for PSMP incorporation of the CdSe/CdS QRs. This finding agrees well with the lack of luminescence of the PSMPs formed upon polymerization of the styrene and divinylbenzene monomers in the presence of the CdSe/CdS QRs discussed in a following section (see also Fig. 7). One plausible explanation for the apparent difficulties to incorporate the QRs into the PSMPs could be the different surface capping ligands, i.e., octadecylphosphonic and hexylphosphonic acid ligands instead of the oleic acid and oleylamine ligands utilized for the stabilization of the core/shell QDs and NPLs. In an earlier work, we hypothesized that the polymer-compatible ligand OBDAC intercalates with the NP capping ligands, and thereby creates a polymer-compatible shell around the NPs^31^. Apparently, the different QR surface chemistry affects the interaction with the OBDAC molecules, thereby reducing the compatibility of the QRs with the polymer matrix formed during the polymerization reaction compared to oleic acid and oleylamine-capped NPs. Possibly, the coverage of the NP organic ligand shell by OBDAC molecules is hampered for octadecylphosphonic acid and hexylphosphonic acid surface ligands compared to oleic acid and oleylamine ligands.

To obtain a first hint on a possible influence of the polymerization reaction parameters on NP spatial distribution within the polymer particles, we compared the spatial distribution of oleic acid/oleylamine capped, OBDAC coated CdSe/CdS QDs (5 ML CdS shell) in PSMPs prepared with two different polymerization protocols. The first is a slightly different polymerization protocol previously reported by us^31^, which was compared with the synthesis of QD stained PSMPs in this study. Interestingly, usage of this previously employed polymerization protocol, from which the polymerization procedure employed in this study was derived, led to CdSe/CdS QDs preferably located in the bead core region. In contrast, the QDs studied in this work are located in the bead surface region, despite the usage of closely matching oleic acid/oleylamine capped CdSe/CdS QDs and OBDAC in both studies. Main differences between the polymerization protocols utilized previously and in this work are the slightly higher stirring speed and the slightly different temperature (100 rpm/80 °C in this work vs. 70 rpm/70 °C previously employed), the shorter reaction time (4 h compared to 24 h), and the smaller amount and differing molecular weight of the surfactant PVP (400 mg with 40,000 g/mol compared to 1.465 g with 58,000 g/mol). Apparently, not only the type of NP, but also the reaction conditions chosen for the polymerization reaction can influence NP incorporation and NP spatial distribution within the PSMPs. An increase in stirring speed and temperature usually leads to smaller polymer particles for this type of dispersion polymerization^31^, but the influence of a surfactant with higher molecular weight has not yet been assessed. As this topic is relatively complex, we plan to explore the reason for the difference in NP location inside the PSMPs in depth in a future study.

### Influence of bead synthesis on the luminescence properties of the NP-stained PSMPs

**Figure 5:**
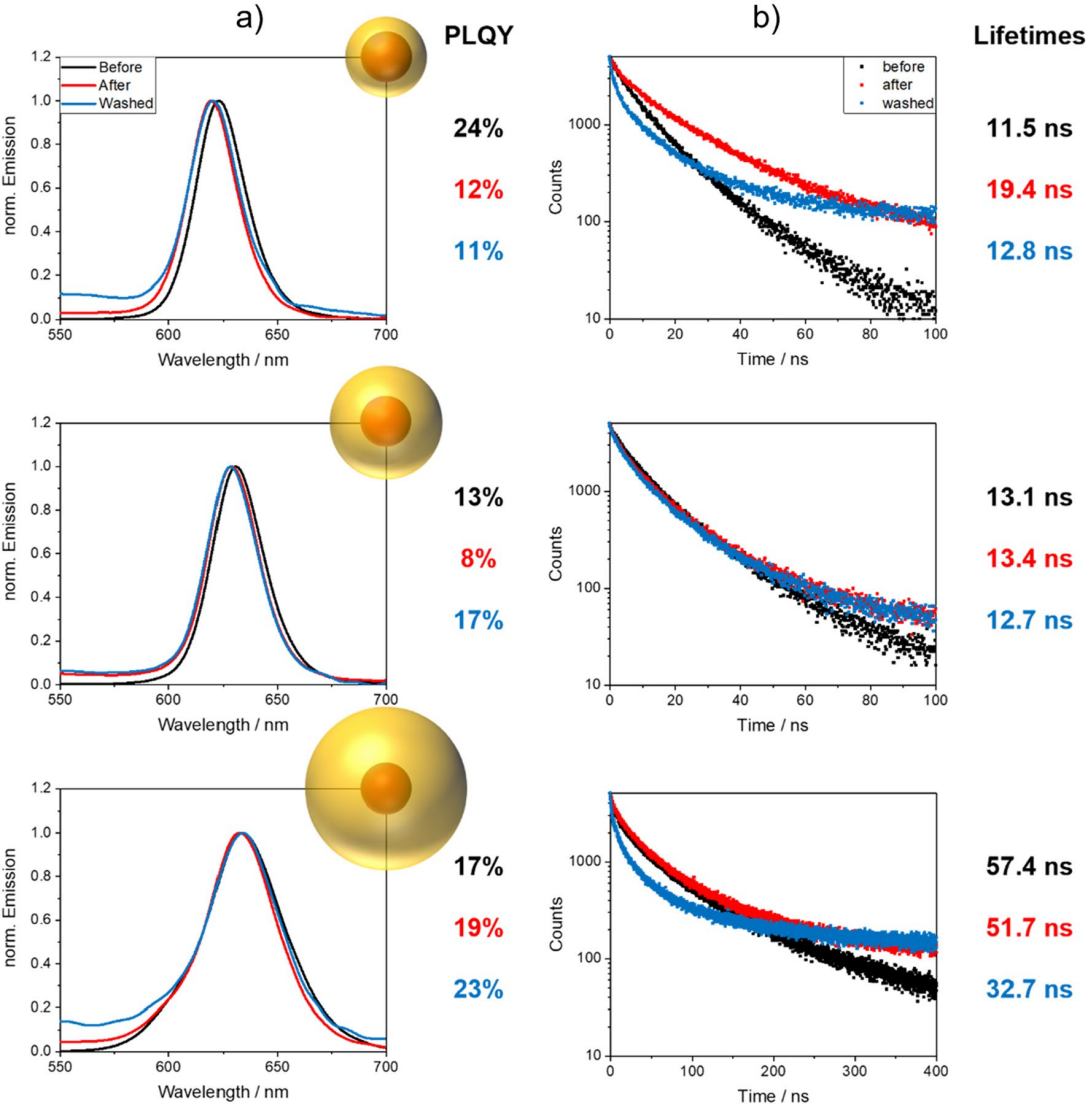
(**a**) Normalized emission spectra with PLQY values, and (**b**) PL decay curves with intensity weighted average FLTs (FLT determination see SI, Eq. SE1) obtained for the NP-stained PSMPs prepared with CdSe/CdS-core/shell QDs of different shell thicknesses (3, 5 and 10 ML from top to bottom). The samples collected before (black) and after (red) bead synthesis were used for the measurements without purification. The “washed” (blue) sample was purified, involving five washing cycles with fresh ethanol. The particle concentration was kept identical by always using the same pipette to remove/add the identical amount of solvent. The higher background in the emission spectra of the washed samples can be attributed to the polymer matrix and scattering effects. The luminescence properties of the analog assessed CdSe/ZnS QDs are displayed in the SI (see Fig. S6).

For the screening study of the influence of the polymerization reaction mixture and the polymerization reaction on the luminescence properties of the different QDs, QRs, NPLs in the resulting NP-stained PSMPs, the polymerization conditions were kept identical. Therefore, for each NP, we spectroscopically characterized the following samples: (i) a sample of the freshly prepared polymerization mixture taken prior to the start of the polymerization reaction; as well as the resulting NP-stained PSMPs after the synthesis (ii) without purification and (iii) after subsequent washing steps. This included measurements of the PL spectra, PLQY values, and PL decay curves from which the intensity weighted average FLTs were calculated (see SI, Eq. SE1). Thereby, information on the application-relevant PL features at different stages of the preparation of the NP-stained PSMPs can be obtained. However, this data does not enable a distinction between the influence of the polymerization reaction and the changes in NP environment, which was beyond the scope of this screening study. The obtained PL data are displayed in Fig. 5 (CdSe/CdS QDs) and Fig. 7 (CdSe/CdS QRs and CdSe/CdS NPLs). An overview of the PL properties, including the PLQY values, of all NPs explored and the respective NP-stained PSMPs subsequently discussed is given in the SI (see Table S1 and Fig. S7).

At first, we explored the influence of the polymerization reaction on the luminescence properties of the resulting NP-stained PSMPs for CdSe QDs, surface passivated with differently thick CdS shells. As follows from Fig. 5, the thickness of the CdS surface passivation shell plays a significant role in the preservation of the optical properties of the CdSe/CdS QDs, with a thicker CdS shell providing a more efficient protection of the emissive CdSe core. This is reflected by the emission spectra and PLQY data shown in the left panels of Fig. 5, showing the highest PLQY for the QDs passivated with a 10 ML CdS shell with a value of 23% compared to 11% and 17% for CdSe/CdS QDs with 3 and 5 ML thick CdS shells. This is remarkable because these 10 ML shell QDs exhibit the lowest PLQY values in hexane of 59%, compared to 78% and 67% observed for CdSe with 3 and 5 ML thick CdS shells (see also Table 1). Time-resolved PL measurements of the different CdSe/CdS QDs in the respective samples reveal that NP incorporation into the PSMPs results in the appearance of a long-lived component in the luminescence decay curves. This change in the PL decay kinetics becomes more pronounced with the progression of the polymerization reaction and bead formation. In the case of the CdSe QDs surface passivated with a 5 ML CdS shell, the observed small influence of the washing steps on the emission intensity and PL decay behavior together with the strong PLQY reduction suggest the presence of so-called “dark” QDs removed during this step, see also next section. This significantly differs from the behavior displayed by the other two CdSe/CdS QDs studied. For these QDs, PL measurements suggest the presence of free QDs, not incorporated into the formed PSMPs, which are still emissive.

The full width at half maximum (FWHM) of the PL bands of the CdSe/CdS QDs surface passivated with 3 ML and 5 ML CdS shells undergo maximum changes of 5% (3 ML QDs: 5%; 5 ML QDs: 4%) during bead formation. In the case of the CdSe/CdS QDs with a 10 ML CdS shell, the FWHM is reduced by about 5% by the polymerization reaction. This points to an incomplete PSMP incorporation of the 10 ML QDs, with the largest QDs of the particle batch remaining in solution; hence, these QDs do not contribute to the PL of the NP-stained PSMPs. This results in a narrowing of the size distribution of the PSMP-encapsulated QDs, and in turn in a smaller FWHM. Combined with the very small shift of the emission maximum, these findings do not provide a hint for a disintegration of the CdS shell. It seems to be more likely that the surface chemistry of the QDs is altered upon incorporation into the polymer matrix, leading to the observed decrease in emission intensity and PLQY. These findings underline the importance of QD surface passivation for the preservation of the PL properties of the PSMP-incorporated NPs, at least for the polymerization conditions chosen for this study.

**Figure 6:**
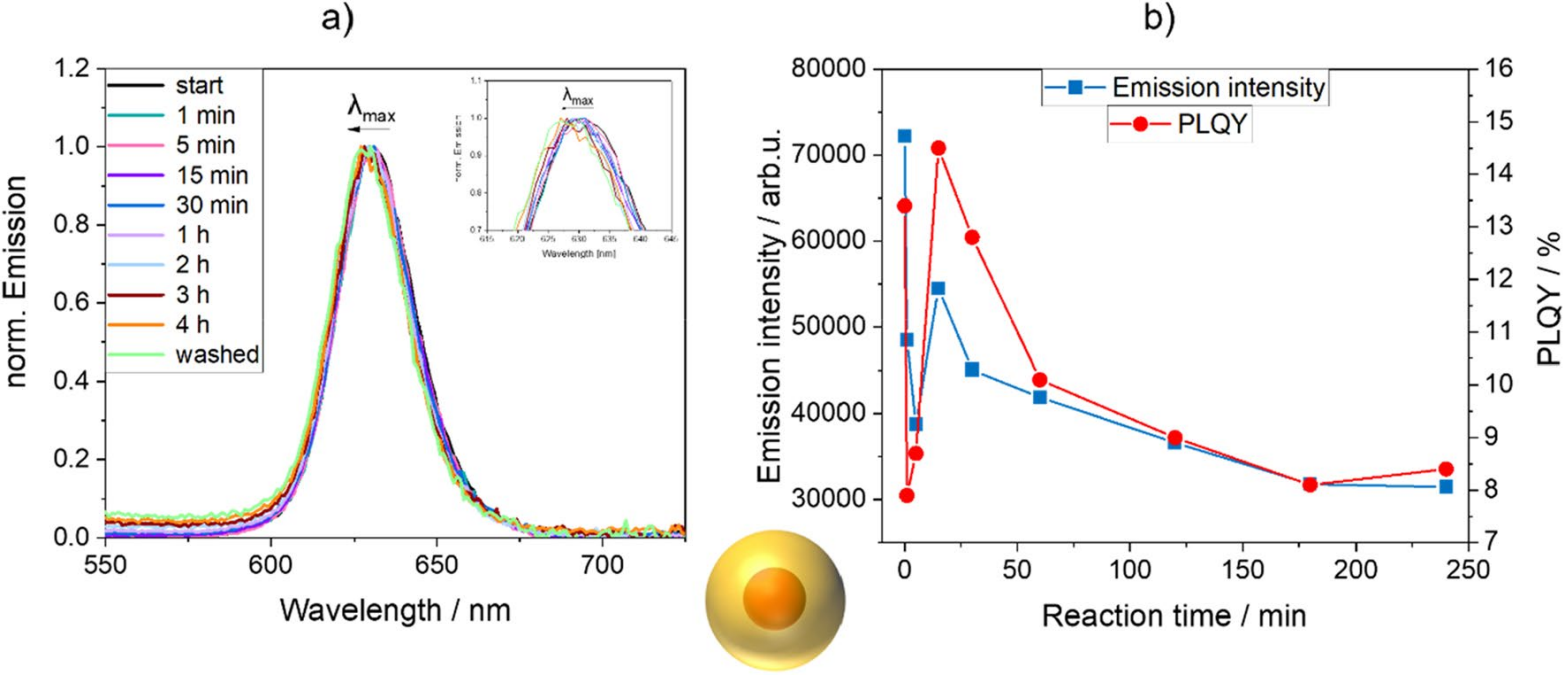
(**a**) Normalized emission spectra of CdSe/CdS QDs passivated with a 5 ML CdS shell before, during, and after incorporation into PSMPs, and (**b**) reaction time-dependent emission intensity and PLQY values of the QDs at the different stages of the PSMP-forming polymerization reaction and in the different microenvironments. Except for the washed QD-stained PSMPs, all samples were taken from the reaction mixture (always using the same amount of the reaction mixture), diluted with fresh ethanol without further purification, and measured immediately to preserve the luminescence properties. The last sample shown in this figure in the left panel a) was purified by five washing steps with fresh ethanol while keeping the particle concentration constant.

For a further in-depth study, monitoring the luminescence properties of the CdSe QDs during the polymerization reaction, we chose CdSe QDs passivated with a 5 ML CdS shell. Thereby, samples were taken from the polymerization mixture with a syringe at different reaction times, diluted with fresh ethanol, and immediately measured to prevent a further influence of the polymerization reaction mixture on the optical properties of the QDs and to ensure sample comparability. The emission spectra and PLQY values of the CdSe/CdS QDs in the samples recorded at different reaction times are summarized in Fig. 6. As shown in this figure, the emission intensity and PLQY values of the QDs immediately decreased in the reaction mixture during the first few minutes of the reaction. Between 5 to 15 min, the emission intensity and the PLQY values of the QDs increased again, reaching approximately the start values of both PL parameters. Presumably, the QDs are incorporated into the PSMPs in this timespan, leading to a change in QD environment and thus, PL properties. The continuous exposure to hot ethanol, and maybe also to the radicals formed from the initiator AIBN at longer reaction times then induce a diminution of the QD PL intensity and the PLQY values at longer reaction times until the polymerization reaction is completed. We assume that the former is mainly responsible for the observed effects, as other researchers reported on a beneficial effect of AIBN on QD PL^58^. The size of the CdSe/CdS QDs did not appear to be altered during the polymerization reaction, since the FWHM of the QD PL band barely changed, varying between 26 and 27 nm as previously mentioned.

In addition to the samples continuously taken during the polymerization reaction, one sample was taken after completion of the polymerization reaction, washed five times with fresh ethanol, and then spectroscopically assessed. To keep the particle concentration constant during these successive washing steps, the same pipette was used to remove/add the identical amount of solvent. The exposure of the NP-stained PSMPs to ethanol during these washing steps was not expected to significantly affect the PL intensity of the QDs, as previously shown by us for a similar sample^31^. Apparently, while the subsequent washing steps do not significantly influence the samples PL maximum and FWHM, the PLQY of the NP-stained PSMPs is increased by more than 100%. This finding points to the presence of dark, i.e., non-emissive, but still light absorbing QDs at the outside of the formed PSMPs, which are removed by the washing steps. To conclusively evaluate this observation, the amount of cadmium in the formed PSMPs could be compared with the amount of initially employed QDs to determine the incorporation efficiency of the QDs. This in turn could help to identify the presence of non-emissive QDs and other NPs in the PSMPs. This was attempted with atomic absorption spectroscopy, see also a previous work^31^, but in our case, we could not obtain conclusive results. We are currently assessing different analytical methods regarding their suitability to reliably provide the NP content of NP-stained PSMPs with a high sensitivity.

**Figure 7:**
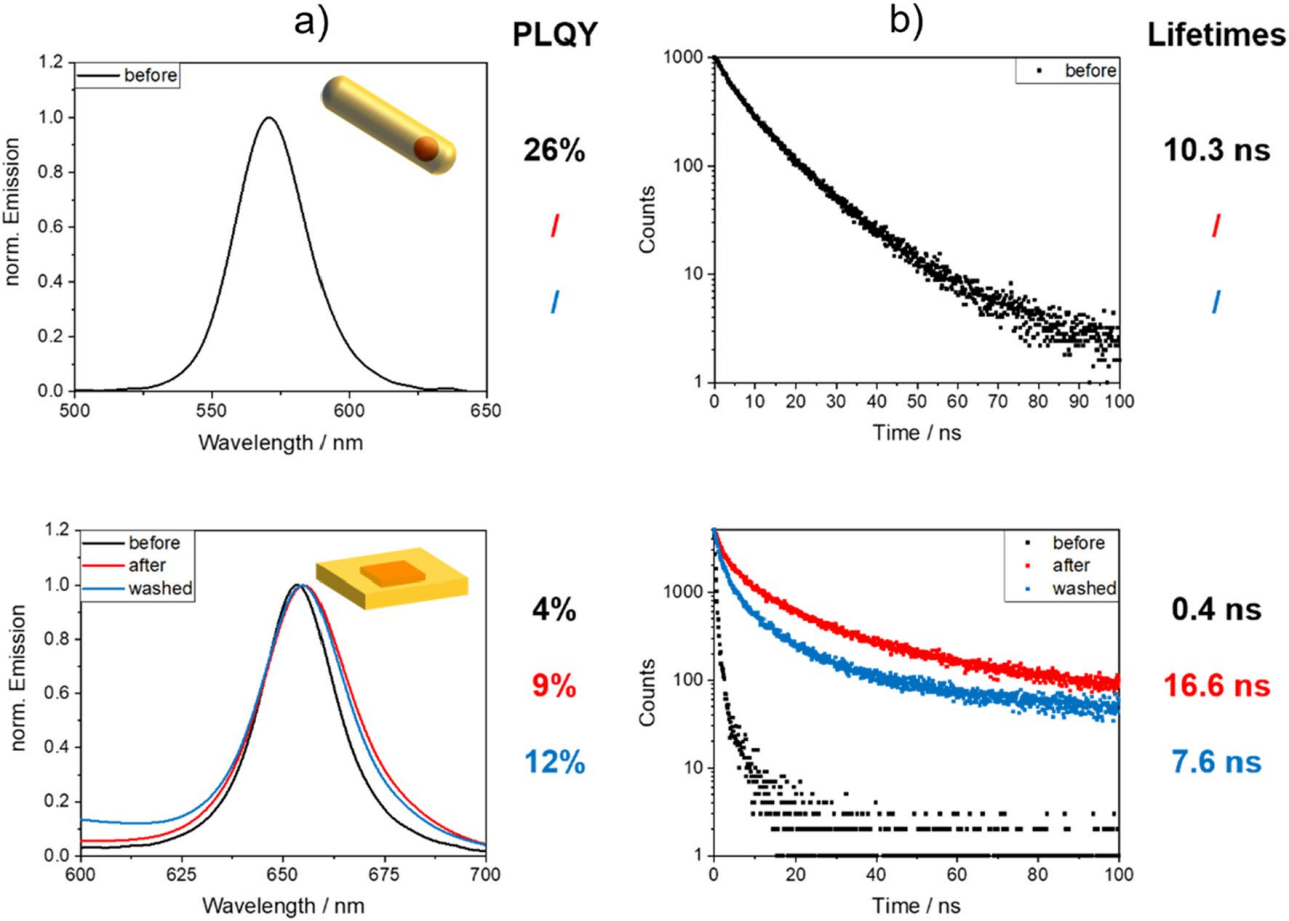
(**a**) Normalized emission spectra of the CdSe/CdS QRs (top) and the CdSe/CdS core/shell NPLs (bottom) before and after the polymerization reaction yielding the respective NP-stained PSMPs, and after bead purification by washing with ethanol including the corresponding PLQY values (left), and (**b**) the corresponding fluorescence decay curves with the intensity weighted average FLT (see SI, Eq. SE1). The samples collected before and after PSMP synthesis were employed for the spectroscopic measurements without purification. The purified samples were washed five times with fresh ethanol, thereby keeping the particle concentration constant. In the case of the QRs, only the purified QR sample exhibited a measurable fluorescence.

Subsequently, we explored the influence of the polymerization reaction and PSMP incorporation on the PL properties of the CdSe/ZnS QDs as well as the CdSe/CdS QRs and CdSe/CdS NPLs. The emission spectra, PLQY values, and PL decay curves of these semiconductor NPs employed for PSMP staining are displayed in Fig. 7 and in the SI in Fig. S6. Apparently, in contrast to the CdSe QDs, surface passivated with a 3 ML CdS shell, which remain emissive during the polymerization reaction and incorporated within the formed PSMPs, the CdSe QDs coated with a 3 ML ZnS shell seem to decompose during the polymerization reaction. This is indicated by the complete loss in PL observed for the PSMPs stained with these QDs. This complete PL quenching, which already occurred in the polymerization mixture before the initiation of the polymerization reaction, can be readily observed with the aid of a hand-held UV flashlight acting as a 365 nm excitation light source, as well as with a spectrometer. It can be speculated from the TEM images of the CdSe/ZnS QDs shown in the SI in Fig. S5, and the deduced particle size of 5.1 nm, that the ZnS passivation shell is thinner than the intended 3 ML thickness. This relatively thin ZnS surface protecting shell is most likely the cause for the observed quenching of the CdSe/ZnS QD fluorescence or at least strongly contributes to it. It has been reported for CdSe/ZnS QDs that the reaction with AIBN radicals can enhance the PL of the QDs, likely by reacting with excess sulfide at the ZnS surface^58^. Also, the successful incorporation of ZnS-shelled NPs into a polymer and successful polymer shelling employing a radical polymerization with AIBN has been realized before^59^. This confirms that such ZnS-shelled NPs can be principally compatible with reactions initiated by radicals. In our case, we speculate that the very thin ZnS shell of our CdSe/ZnS QDs degraded too fast, possibly due to ligand detachment, which could have favored the oxidation of the thereby exposed core in the ethanolic reaction mixture and harsh reaction environment, leading to PL quenching by formed surface defects.

Our findings demonstrate the crucial influence of the shell material, which passivates and protects the surface of the emissive QD core, on the preservation of the initial QD PL utilizing a dispersion polymerization reaction to synthesize NP-stained PSMPs. For the staining of PSMPs with luminescent CdSe/ZnS QDs employing these reaction conditions, a further optimization of the ZnS shell seems to be necessary. Although a thicker ZnS shell could provide a better protection of the emissive QD cores and their PL properties, an increase in shell thickness can also lead to initially lower PLQY values of the CdSe/ZnS QDs^60^. A more promising approach for this type of CdSe QDs could be the usage of milder reaction conditions for the preparation of luminescent, NP-stained PSMPs that have to be identified in screening studies.

The CdSe/CdS QRs assessed, which exhibit a moderate emission intensity and PLQY as shown in Table 1, become barely luminescent or non-emissive after the polymerization reaction and presumed incorporation into the PSMPs. Examination of the residue of the polymerization reaction reveals the presence of large, luminescent polymer agglomerates at the bottom of the flask. This indicates an incomplete incorporation of the QRs into the beads. With the aid of STEM measurements shown in Fig. 3, we could demonstrate that the QRs are not incorporated into the PSMPs. These findings suggest that the surface chemistry of the CdSe/CdS QRs capped with octadecylphosphonic acid/hexylphosphonic acid ligands and not with the oleic acid/oleylamine ligand shell utilized for all other semiconductor nanostructures explored in this study is not suitable for the polymerization protocol employed by us. In the future, we plan to systematically examine the influence of the QD ligand shell and its composition on the incorporation efficiency of different semiconductor nanostructures into polymer beads utilizing our established polymerization protocol.

For the CdSe/CdS core/shell NPLs, the polymerization reaction mixture has a significant influence on the PL properties of these semiconductor nanostructures, even before the initiation of the polymerization. This influence is, however, reduced upon the incorporation of the NPLs into the PSMPs. The observed decrease in emission intensity after the washing steps employed for purification suggests that not all NPLs present in the polymerization mixture are incorporated into the PSMPs formed. In addition, the polymerization reaction induces a slight broadening of the NPL emission band by about 17%. These findings, combined with the very small shift of the emission maximum, suggest that the NPL size/thickness is not affected by the polymerization reaction, but instead the NPL surface chemistry and/or the number of surface defect states. For these fragile semiconductor nanostructures, the relatively high degree of preservation of the initial PLQY value of the NPLs in hexane of 34% (see Table 1), yielding a PLQY value of 12% for the NPLs incorporated into PSMPs (see Fig. 7, lower panel), is very promising. The PL decay kinetics of the PSMP-incorporated NPLs exhibit a longer decay component than previously observed for the initially prepared NPLs. A similar behavior was also observed for the CdSe/CdS QDs. This points to defect emission of the NPLs. In addition, the faster FLT components of the PL decay kinetics of the CdSe/CdS NPLs increase after PSMP incorporation. As the NPL luminescence is reduced, but still well detectable, this suggests that most likely only the outside of the CdS shell or the ligand layer is affected by the polymerization reaction.

## Conclusion and outlook

We performed a screening study of the incorporation of different, luminescent CdSe-based semiconductor nanoparticles (NPs) varying in size, shape, and particle composition into polystyrene microparticles (PSMPs) by a radical polymerization reaction, thermally induced in the presence of the NPs. Nanostructures representatively explored included spherical core/shell CdSe quantum dots (QDs) surface passivated with differently thick CdS shells and for comparison, also with a ZnS shell. In addition, elongated core/shell CdSe/CdS dot-in-rods, or so-called quantum rods (QRs), as well as core/shell CdSe/CdS nanoplatelets (NPLs) as representative 2D-nanostructures were assessed. For the preparation of all NP-stained PSMPs, the same polymerization conditions were employed, which were adapted from previously optimized synthesis protocols. Subsequently, the size, size distribution, and surface morphology of the NP-stained PSMPs and the spatial distribution of the NPs within the polymer beads were determined by electron microscopy and correlated with NP properties. NP properties examined included size, shape, and particle architecture, i.e., chemical composition and thickness of the surface passivation shell and the chemical composition of the organic ligand shell. As parameters for the evaluation of how and to which extent the size, shape, and architecture of the CdSe-based nanostructures affect the application-relevant luminescence properties of the resulting NP-encoded beads, we utilized the polymerization-induced changes in the PL maximum, PL spectral width, PL quantum yield (PLQY), and PL decay kinetics of the initially prepared NPs. Special emphasis was dedicated to the degree of preservation of the NP PLQY.

The importance of NP surface chemistry is underlined by the straightforward incorporation of spherical and platelet-shaped CdSe/CdS-core/shell NPs surface capped with oleic acid and oleylamine ligands, while the incorporation of cylindrically shaped NPs like QRs of the same core/shell composition, but stabilized with octadecylphosphonic and hexylphosphonic acid molecules, failed for our polymerization protocol. The considerable influence of the NP surface ligand shell seems to be directly associated with the degree of coverage of the organic ligand shell initially present from NP synthesis by OBDAC molecules, which were added to assure NP compatibility with the polymer matrix. In the future, we plan a thorough assessment of the NP ligands before and after the coating with OBDAC. Also, to confirm our hypothesis of the importance of NP surface chemistry, the incorporation of QRs capped with oleic acid and oleylamine ligands will be examined. The spatial distribution of the NPs within the PSMPs suggests that for the polymerization protocol used, the NPs do not act as seeds for the polymerization reaction and are not incorporated into the polymer particle seeds formed at the initial stage of the polymerization reaction, yet at a later stage of seed growth.

Our PL studies show that for the preservation of the initial PL of the semiconductor nanostructures during the thermally induced dispersion polymerization reaction, at least for the reaction conditions employed in this study, the shell material, passivating and protecting the surface of the emissive QD core, and the shell thickness are crucial parameters. For core/shell CdSe QDs, a CdS surface protection shell is apparently superior to a ZnS shell for the preservation of the PL properties, as revealed by the complete PL loss of CdSe/ZnS with a thin ZnS shell for the chosen polymerization conditions. For surface passivation with CdS shells, shell thickness clearly matters, with a thicker shell being advantageous for high PLQY of the PSMP-incorporated QDs. The effect of the shell thickness on the PL preservation is not unexpected but has still not been reported before for NP-stained PMPs.

Promisingly, the most sensitive nanostructures assessed in this study, CdSe/CdS NPLs, survived the harsh polymerization conditions, and were successfully encapsulated into polymer beads while remaining emissive. Particularly favorable is the relatively high percentage of preservation of the initial PLQY value of the NPLs observed for the PSMP-incorporated NPLs. This degree of PLQY preservation equals or even exceeds the changes in PLQY observed for the much more robust CdSe/CdS QDs. This demonstrates that both spherical and non-spherical semiconductor NPs with appropriate surface chemistry can be incorporated into PMPs with our synthesis procedure. These findings form the basis for a synthesis approach that is simple and suitable for a broad spectrum of luminescent NPs. Encouraged by the outcome of this first screening study, the ultimate goals, should be the precise control of the spatial distribution of the NPs within the resulting polymer beads and optimized PL preservation for different sets of semiconductor nanostructures with fine-tuned surface chemistry. In the future, we also plan to monitor the different stages of the polymerization reaction in the presence of several NPs in more detail to derive a better understanding of the role of the NPs in the polymerization reaction and obtain improved mechanistic insights.

### Supplementary Information

Supplementary Information.

## Data Availability

All data generated/analyzed during this study are included either in this article and its Supplementary Information files or are available upon request to the corresponding author (U. Resch-Genger, ute.resch@bam.de) or the first author (L. Scholtz, lena.scholtz@bam.de).
