# Supplementary material for: Correlating semiconductor nanoparticle architecture and applicability for the controlled encoding of luminescent polymer microparticles
Source: Sci Rep. 2024 May 24;14:11904. doi: 10.1038/s41598-024-62591-1 (PMC11126414; doi:10.1038/s41598-024-62591-1)
Supplement: Supplementary file 1 — Supplementary Information. [file 41598_2024_62591_MOESM1_ESM.pdf]

Supplementary Information for:

Correlating Semiconductor Nanoparticle Architecture and Applicability for the Controlled Encoding of Luminescent Polymer Microparticles

Lena Scholtz[1,2], J. Gerrit Eckert[3,4], Rebecca T. Graf[3,4,5], Alexandra Kunst[1,2], K. David Wegner[1], Nadja C. Bigall[3,4,5], Ute Resch-Genger[1,*]

[1]Federal Institute for Materials Research and Testing (BAM), Division 1.2 *Biophotonics*, Richard-Willstätter-Str. 11, 12489 Berlin, Germany.

[2]Free University Berlin, Institute for Chemistry and Biochemistry, Takustraße 3, 14195 Berlin, Germany.

[3]Leibniz University Hannover, Institute of Physical Chemistry and Electrochemistry, Callinstraße 3A, 30167 Hannover, Germany.

[4]Cluster of Excellence PhoenixD (Photonics, Optics, and Engineering − Innovation Across Disciplines), 30167 Hannover, Germany.

[5]Leibniz University Hannover, Laboratory of Nano- and Quantum Engineering, Schneiderberg 39, 30167 Hanover, Germany.

*ute.resch@bam.de, Phone: +49 (0)30 8104 1134

1. Synthesis of CdSe/CdS and CdSe/ZnS-core/shell-QDs 2

2. Synthesis of CdSe/CdS-core/shell-NPLs 2

3. Preparation and ^{1}H-NMR spectrum of polyethylene glycol-*block*-poly(ε-caprolactone) 3

4. Fluorescence lifetime decay curves of all NPs and calculation of FLTs 4

5. Size distribution graphs of NPs and NP-stained PSMPs 5

6. TEM and SEM images of remaining, employed QDs and respective PSMPs 7

6. Emission spectra of CdSe/ZnS QDs and the respective, stained PSMPs 7

7. Preservation of NP luminescence properties upon integration into the PSMPs 8

8. STEM images of PSMPs stained with QDs from previous works 9

1. Synthesis of CdSe/CdS and CdSe/ZnS-core/shell-QDs

The CdSe/CdS-core/shell-QDs with shell thicknesses of about 3, 5, and 10 monolayers (ML) were prepared from the same CdSe core particles according to a modified synthesis adapted from Carbone *et al.*, Nightingale *et al.* and Chen *et al.*,[1-3] which was partly previously described by us.[4,5] The same CdSe cores were also employed for the synthesis of CdSe/ZnS-QDs with a ZnS shell thickness of approximately 3 ML. The shell growth was carried out according to a self-developed procedure by J. G. Eckert.

In the first step, CdSe cores with wurtzite structure were synthesised according to Carbone *et al.*[6] For this synthesis, 120 mg (0.93 mmol) CdO together with 560 mg (1.67 mmol) ODPA and 6 g (15.51 mmol) TOPO were degassed at 150 °C for 1 h. The mixture was then heated under argon flow to 300 °C. After the injection of 2 mL (4.48 mmol) of TOP, it was heated to 380 °C and, following a retention period of 10 min, 3.6 mL of a previously prepared TOP/Se solution (120 mg/3.6 mL) was swiftly injected. The temperature was allowed to rise to 380 °C again before the reaction was quenched by addition of 5 mL of ODE and cooled down to 70 °C in an air stream. During the cooldown period, 5 mL of toluene was added to prevent solidification. The resulting particles were precipitated by methanol/isopropanol (1:2), centrifuged at 6,000 rcf and redispersed in 2 mL of hexane.

A $Cd(oleate)_2$ precursor solution was synthesised according to Nightingale *et al.*[7] For this synthesis, a mixture of 1.284 g (1 mmol) CdO, 12.94 mL (40.77 mmol) of oleic acid and 7.04 mL of ODE was degassed for 10 min at 100 °C. The dispersion was heated to 180 °C under argon flow and kept there for 60 min under vigorous stirring. To remove water as a side product, the mixture was cooled to 120 °C and degassed for 45 min. The 0.5 M $Cd(oleate)_2$ solution was used as prepared for the shell growth step.

$Zn(oleate)_2$ was prepared by using a synthesis procedure described by Boercker *et al.*[8] For this, Zn(acetate)2 and OA (molar ratio 1:4) were mixed in a flask and heated under vacuum to 110 °C while stirring. The reaction was allowed to proceed for 2 h before the mixture was left to cool to RT. The resulting solid was washed with acetone and centrifuged three times at 6000 rcf for 10 min. The final product was dried in a Schlenk flask and stored under argon until further use.

The growth of the CdS surface passivation shell was performed according to an adapted synthesis by Chen *et al.*[9] For this, 100 nmol of the CdSe cores (142.4 µL) were dispersed in 3 mL of ODE and OLA, respectively. The mixture was carefully degassed for 30 min at 90 °C. In the meantime, the S and Cd precursor solutions were prepared. For the different shell thickness of 3, 5 and 10 monolayers, 398/875/3101 µL of $Cd(oleate)_2$ were diluted to a total volume of 7 mL with ODE, respectively. Additionally, 35.7/78.4/278 µL of 1-octanethiol were similarly diluted to a total volume of 7 mL with ODE. The flask was then heated under argon flow in two steps to 310 °C. When reaching 240 °C, the simultaneous injection of the previously prepared $Cd(oleate)_2$ and 1-octanethiol solutions via syringe pump (6 mL, 3 mL/h) was initiated. After two hours, 1 mL (3 mL for 10 ML QDs) of oleic acid was injected, and the temperature was kept at 310 °C for another hour (3 h for 10 ML QDs). Finally, the reaction mixture was cooled down to RT in an air flow, and the particles were precipitated by addition of acetone and centrifuged once before redispersion in hexane. The purification process was not performed more than once because the repeated precipitation and centrifugation of these QDs has led to significant issues with QD redispersion in the past. This occurs due to ligand detachment at low concentrations and subsequent aggregation of the QDs.

The growth of the ZnS shell was performed similarly. The same amount of CdSe cores (142.4 µL) was employed, and all other synthesis parameters were kept the same apart from the amount of 1-octanethiol (40.2 µL, diluted with ODE to 7 mL) and $Zn(oleate)_2$ (140.9 mg, suspended in 7 mL ODE). The solubility of the $Zn(oleate)_2$ in ODE was poor, so the amount that was actually added to the synthesis was presumably slightly lower.

The concentrations of the resulting QD dispersion were determined by AAS (Cd content). They were determined to be 7.88/3.19/32.85 mg/mL for the CdSe/CdS QDs, and 1.30 mg/mL for the CdSe/ZnS QDs.

2. Synthesis of CdSe/CdS-core/shell-NPLs

The $Cd(myristate)_2$ precursor solution was prepared according to Tessier *et al.*[10] For this, $Cd(NO_3)_2 \cdot 4\ H_2O$ (3221 mg) was dissolved in methanol (80 mL). Simultaneously, Na(myristate) (6262 mg) was dissolved in methanol (500 mL) through stirring for 1.5 h. The $Cd(NO_3)_2$ solution was slowly added to the Na(myristate) solution. The resulting $Cd(myristate)_2$ (white precipitate) was washed with 1.5 L methanol in a Buchner vacuum flask. Finally, the $Cd(myristate)_2$ was dried under vacuum for at least 12 h and stored in a glove box afterwards.

The synthesis of the quasi-quadratic CdSe core NPLs with a thickness of 4.5 ML were synthesized according to Abécassis *et al.* and Miethe *et al.*[11,12] First, $Cd(myristate)_2$ (1360 mg), Se powder (108 mg) and ODE (120 mL) were mixed in a 250 mL three-neck round flask for 10 s in an ultrasonic bath. The flask was degassed for 30 min at 70 °C, purged with nitrogen and degassed again for 30 min at 70 °C. The temperature was set to 240 °C under

a nitrogen flow. At 202 °C, the septum was withdrawn, and Cd(acetate)$_2$·2 H_2O (640 mg) was added swiftly to the reaction mixture. The reaction was held at 240 °C for 8 min before OA (4 mL) was injected. The solution was rapidly cooled with compressed air from the outside and OA (4 mL) was added a second time at around 160 °C. The red dispersion was transferred to four centrifuge tubes. Ethanol (in total 50 mL) was added before centrifuging at 4226 rcf for 10 min. The precipitate was redispersed in hexane (in total 60 mL) and centrifuged again at 4226 rcf for 10 min. The supernatant was transferred to new centrifuge vials and precipitated with ethanol (20 mL in total). The dispersion was centrifuged a third time at 4226 rcf for 10 min. The resulting precipitate was redispersed in approximately 8 mL hexane.

To synthesize the CdSe/CdS-core/shell-NPLs, a procedure published by Rossinelli *et al.* was employed and slightly varied.[13] For this, Cd(oleate)$_2$ (270.3 mg), ODE (10 mL), and the CdSe core NPLs dispersed in hexane (438 µL, c(Cd)=39.4 mmol/L) were combined and degassed for about. 20 min at RT, 30 min at 60 °C and 3 h at 80 °C. After the addition of OLA (2 mL, directly from the glove box), the flask was heated to 300 °C under argon flow with about 15 °C/min. At 180 °C, 4.5 mL of a 1-octanethiol/ODE solution (77 µL in 5.5 mL) was injected with a rate of 3 mL/h. Then, the reaction flask was cooled to RT. The reaction solution was transferred to a centrifuge vial, and hexane (5 mL) and ethanol (7.5 mL) were added. The mixture was centrifuged at 4226 rcf for 10 min. The precipitate was redispersed in hexane (6 mL) and centrifuged again at 4226 rcf for 10 min. The red supernatant was again centrifuged in a new vial to get rid of remaining CdO impurities.

Similar to the QDs, the concentration of the NPLs was determined by AAS to be 0.96 mg/mL (Cd content).

3. Preparation and ^{1}H-NMR spectrum of polyethylene glycol-*block*-poly(ε-caprolactone)

The *block*-copolymer polyethylene glycol-*block*-poly(ε-caprolactone) (PEG-*b*-PCL) was prepared according to a previously reported procedure[4] adapted from Meier *et al*.[14]
800 mg of poly(ethylene glycol) were placed in a dry flask with 1536 µL (14.53 mmol) of ε-caprolactone. The mixture was put in a preheated aluminium heating block and stirred for 5 min at 130 °C, before one drop of Sn(II) 2-ethylhexanoate was added as a catalyst and initiator. The reaction mixture was then stirred for 3 h at 130 °C before rapidly cooling it with an ice bath, which lead to the precipitation of a solid, white product. The raw product was recrystallized by dissolving it in a small amount of dichloromethane, followed by precipitation with *n*-heptane. The PEG-*b*-PCL was then filtered and washed several times with *n*-heptane before it was dried.
Characterization of the synthesized PEG-*b*-PCL was performed by nuclear magnetic resonance spectroscopy (solution ^{1}H-NMR) at RT with a 400 MHz JEOL JNM-ECX400 spectrometer (Free University Berlin), the resulting spectrum is displayed in Figure S1. The sample was prepared by dissolving 6 mg of PEG-*b*-PCL in 700 µL of $CDCl_3$.

Chemical shifts: ^{1}H-NMR ($CDCl_3$, 400 MHz): δ = 1.39 (m, 2H, γ), 1.63 (m, 4H, β & δ), 2.30 (m, 2H, α), 3.63 (s, 4H, a & b), 4.05 (t, 2H, ε), 4.21 (t, 2H, b).

According to Meier *et al.*, the number-average molecular weight M_n of the synthesized PEG-*b*-PCL was determined to be about 10,050 g/mol (from the ratio of protons corresponding to the PEG and PCL signals).[14]

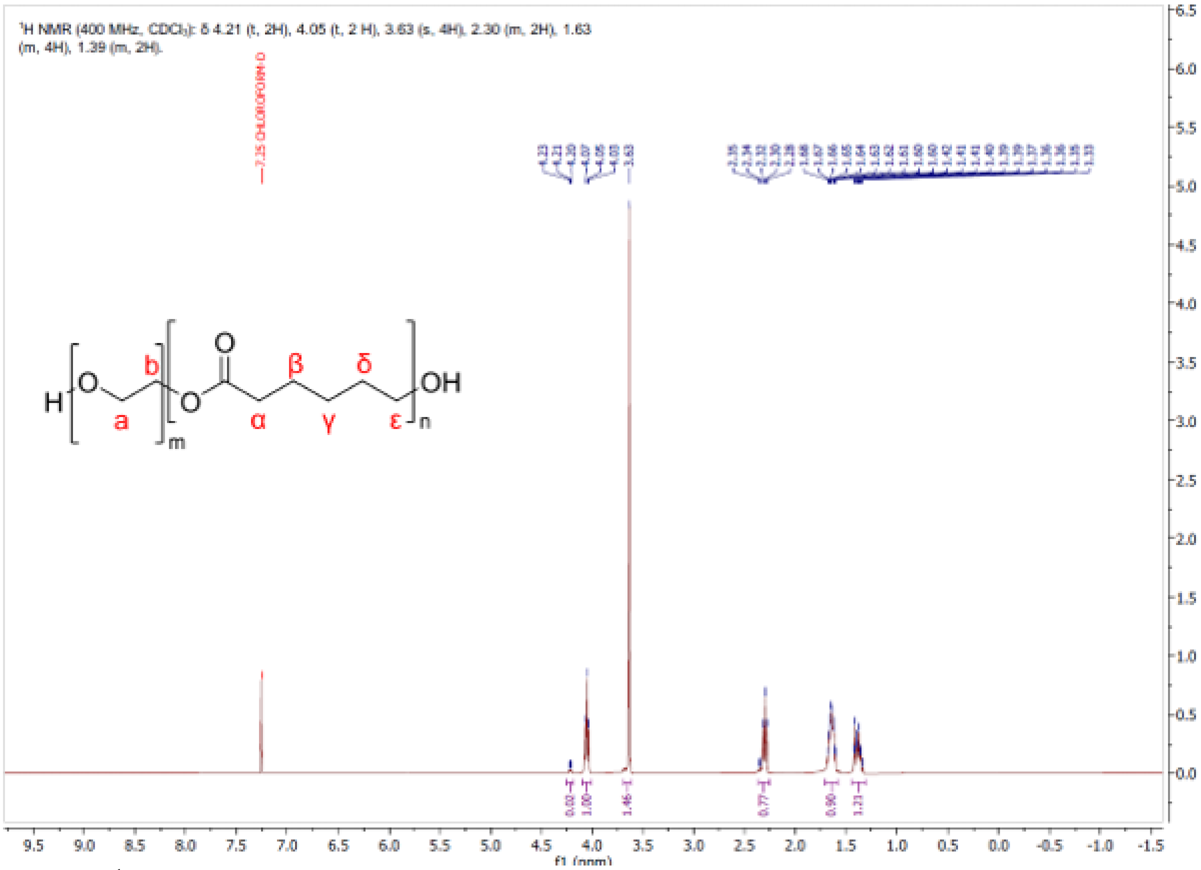

Figure S1: ^{1}H NMR spectrum (400 MHz, solvent $CDCl_3$) with structural formula of PEG-*b*-PCL, including all compound peaks and the solvent peak.

4. Fluorescence lifetime decay curves of all NPs and calculation of FLTs

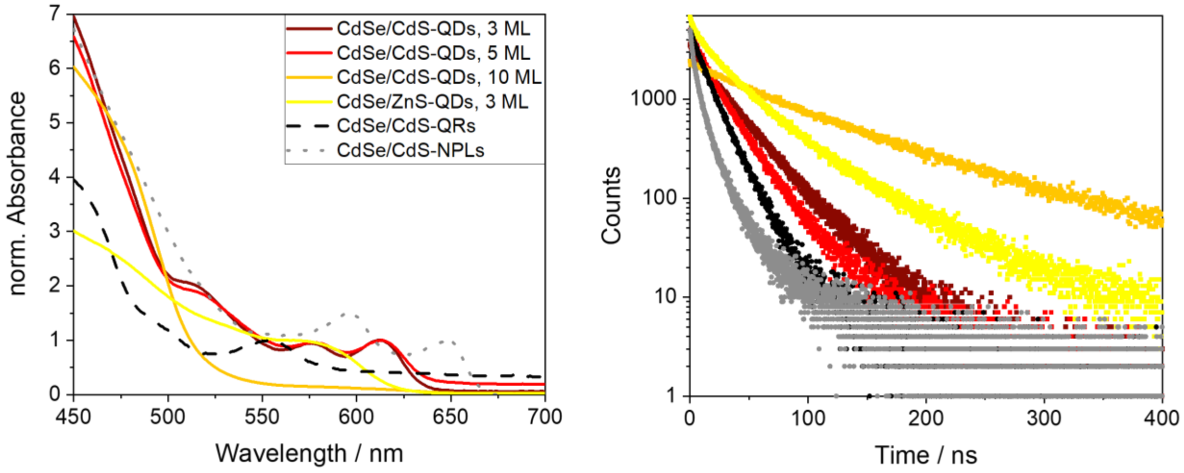

Figure S2: Normalized absorbance spectra (left) and fluorescence decay curves (right) of all employed NPs in hexane.

In the following equations, the intensity average FLT τ_{int} (SE1) and the multi-exponential model (two or three exponents were chosen, depending on the measured NP) for the calculation of component FLTs τ_i from multi-exponential decay curves (SE2) are specified. Here, B_i is the pre-exponential factor of the lifetime component i, and $I(t)$ is the fluorescence intensity as a function of time t.

$$\langle\tau_{int}\rangle = \frac{\sum_{i=1}^{n} B_i \tau_i^2}{\sum_{i=1}^{n} B_i \tau_i} \quad \text{(SE1)}$$

$$I(t) = \sum_{i=1}^{n} B_i exp\left(\frac{-t}{\tau_i}\right) \quad \text{(SE2)}$$

5. Size distribution graphs of NPs and NP-stained PSMPs

In Figure S3 and Figure S4, the size distribution graphs and mean particle sizes of all employed NPs and the resulting, NP-stained PSMPs are displayed.

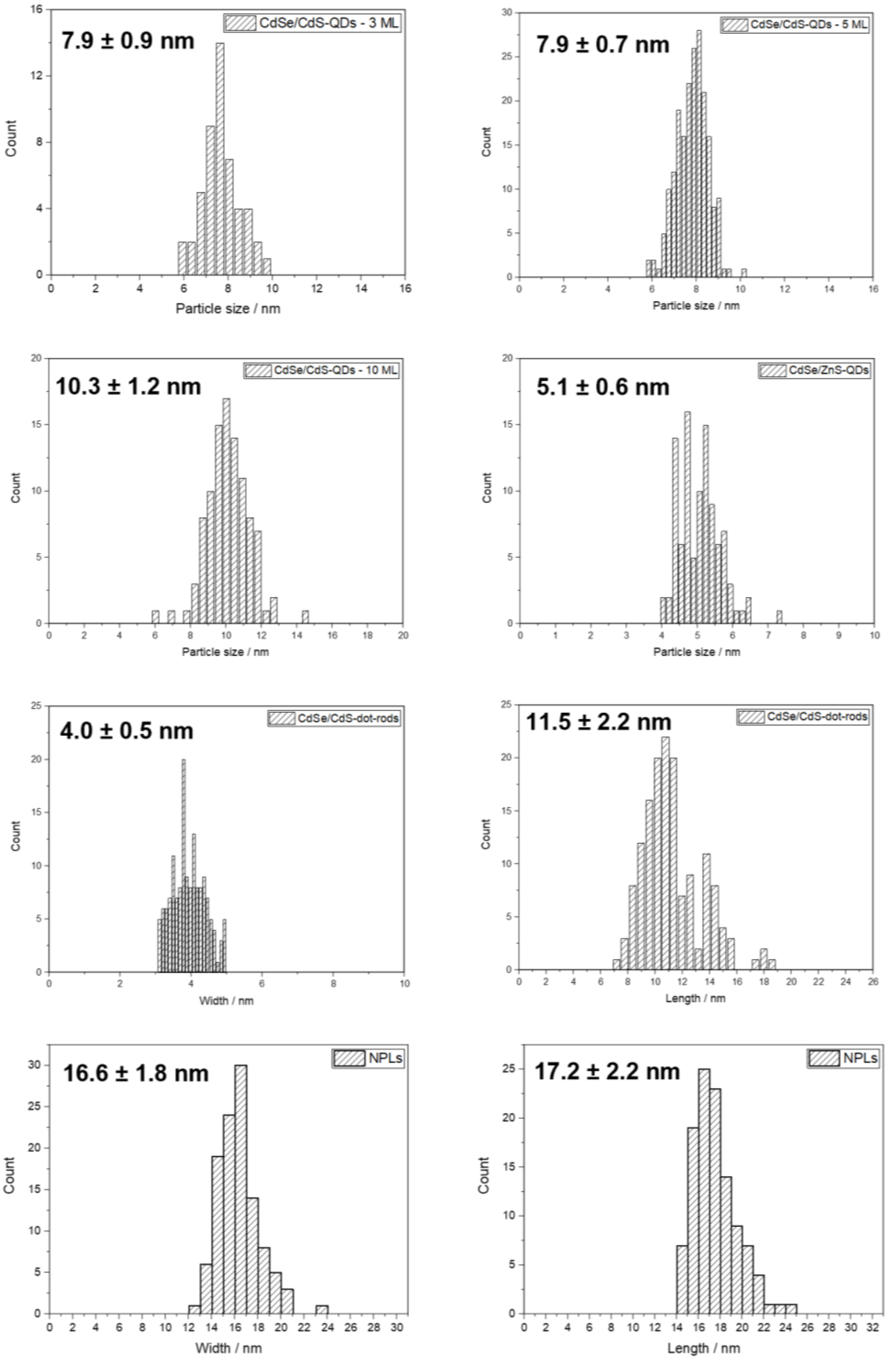

Figure S3: Size distribution histograms for all employed NPs with mean particle size and standard deviation, all sizes determined from TEM images.

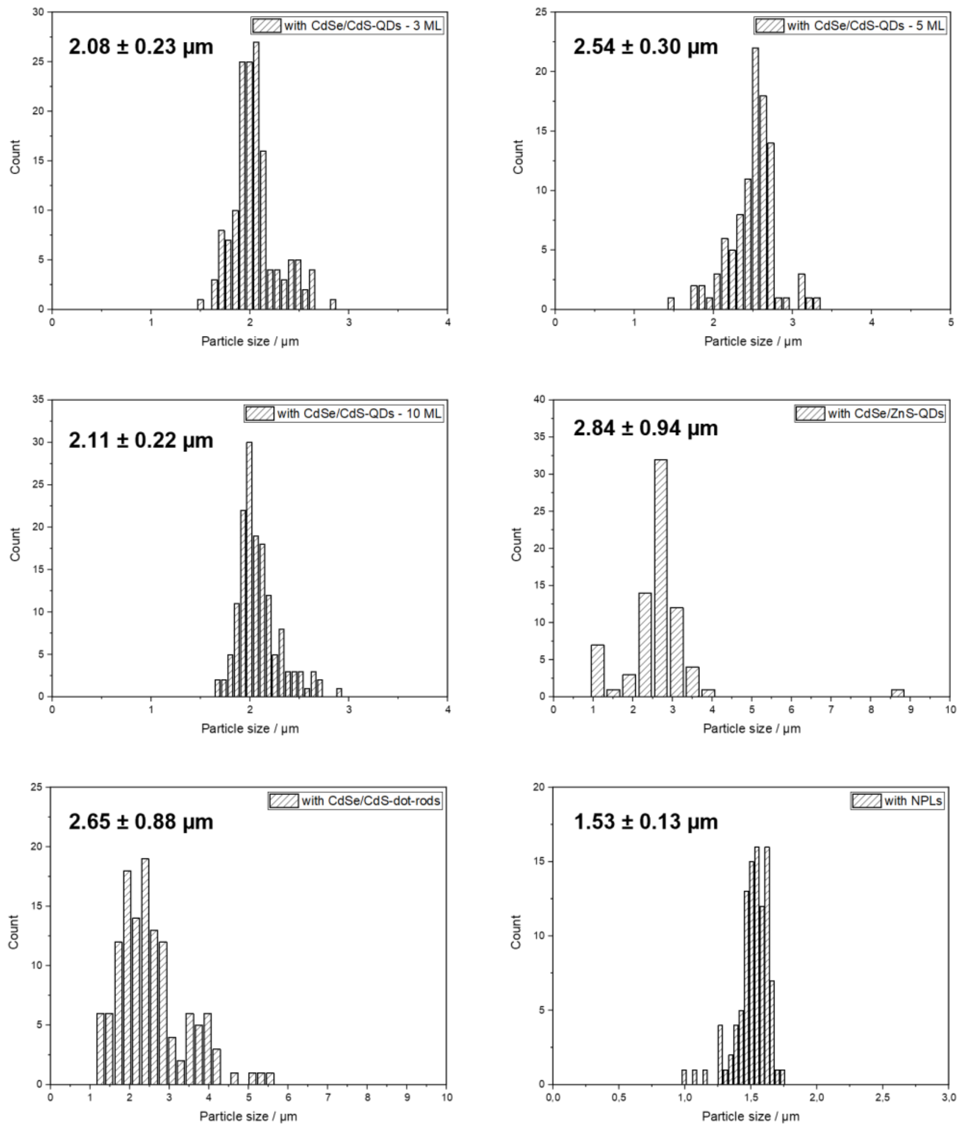

Figure S4: Size distribution histograms for all synthesized, NP-stained PSMPs with mean particle size and standard deviation, all sizes determined from SEM images.

6. TEM and SEM images of remaining, employed QDs and respective PSMPs

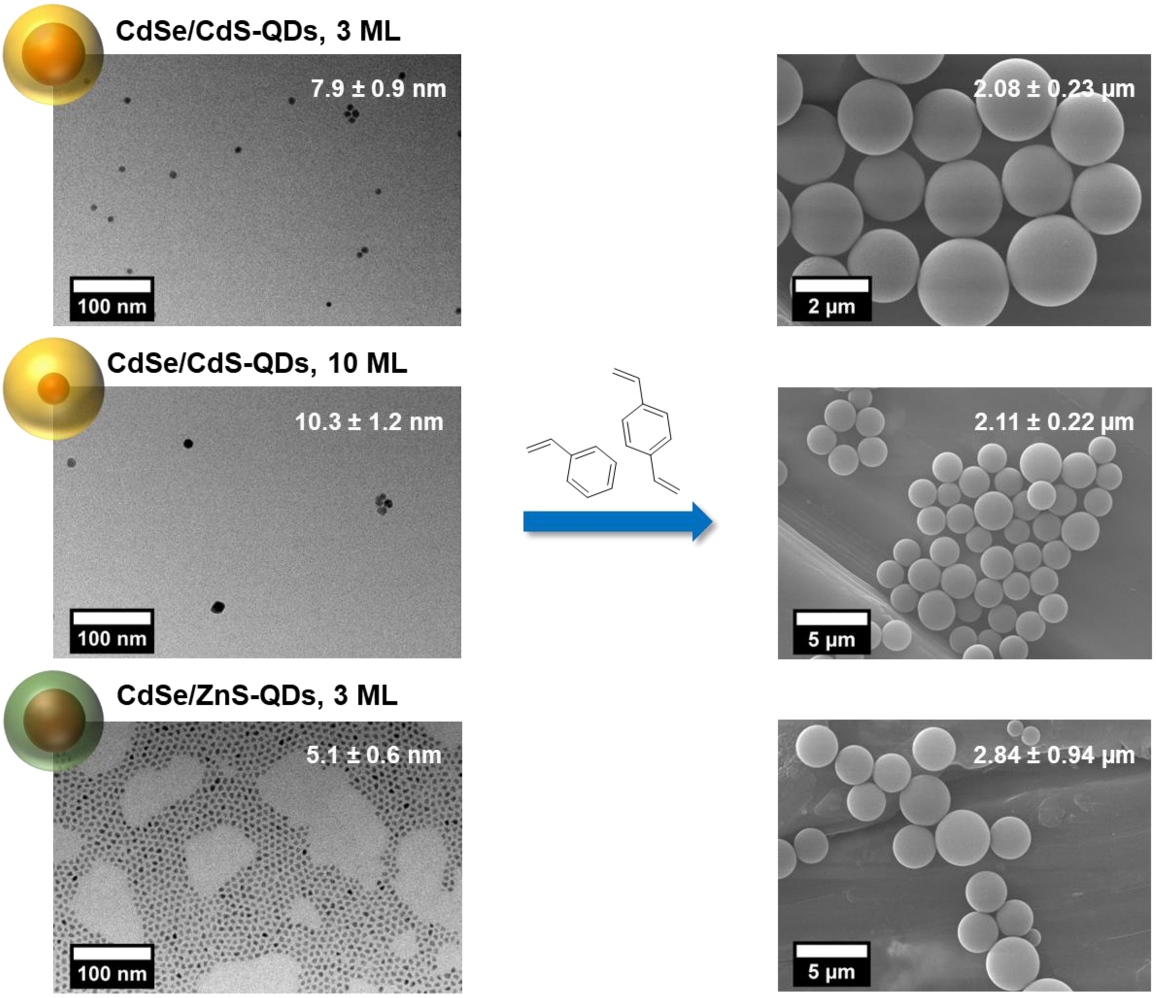

Figure S5: TEM images (left) of CdSe/CdS and CdSe/ZnS QDs, showing the average particle sizes; and the SEM images (right) of the resulting, NP-stained PSMPs with average particle sizes (right).

6. Emission spectra of CdSe/ZnS QDs and the respective, stained PSMPs

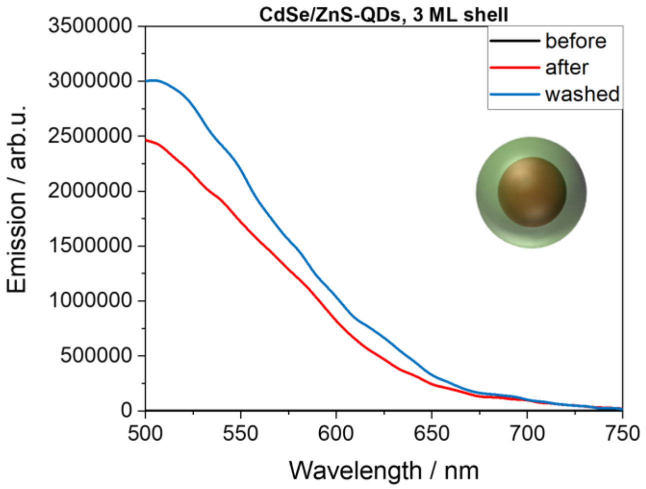

Figure S6: Emission spectra of CdSe/ZnS QDs before, during and after the incorporation into PSMPs. No emission bands of the QDs could be detected, even at the start of the reaction, which points to the destruction/quenching through the reaction mixture.

7. Preservation of NP luminescence properties upon integration into the PSMPs

In Table S1 and Figure S7, the position of the emission maxima, as well as the emission intensity and PLQY preservation of all NPs incorporated into the respective PSMPs are summarized. The preservation is specified as the percentage of emission intensity (P_{em}) or PLQY (P_{PLQY}) of the NPs retained, compared to the respective data before the start of the synthesis. The shift of the emission maximum, induced by the changed environment of the NPs in the polymer matrix, is typically very low (up to 7 nm).

Table S1. Emission maxima and intensity preservation (P_{em}), as well as PLQY values and PLQY preservation (P_{PLQY}), for the PSMPs stained with the different luminescent semiconductor NPs. For the CdSe/ZnS QDs, no remaining emission, and thus no PLQY and FLT, was detected.

**Incorporated NPs**	$\lambda_{em, max}$ **/ nm**	P_{em} **/ %**	**PLQY / %**	P_{PLQY} **/ %**
CdSe/CdS QDs, 3 ML	Hexane: 622	/	78	/
	Start: 623	100	24	100
	End: 630	73	12	50
	Washed: 620	24	11	46
CdSe/CdS QDs, 5 ML	Hexane: 627	/	67	/
	Start: 631	100	13	100
	End: 627	44	8	62
	Washed: 627	38	17	131
CdSe/CdS QDs, 10 ML	Hexane: 638	/	59	/
	Start: 634	100	17	100
	End: 632	93	19	112
	Washed: 634	35	23	135
QRs	Hexane: 566	/	86	/
	Start: 571	100	26	100
	End: 573	2	2	8
	Washed: 566	3	2	8
NPLs	Hexane: 656	/	34	/
	Start: 653	100	4	100
	End: 655	1951	9	225
	Washed: 655	758	12	300

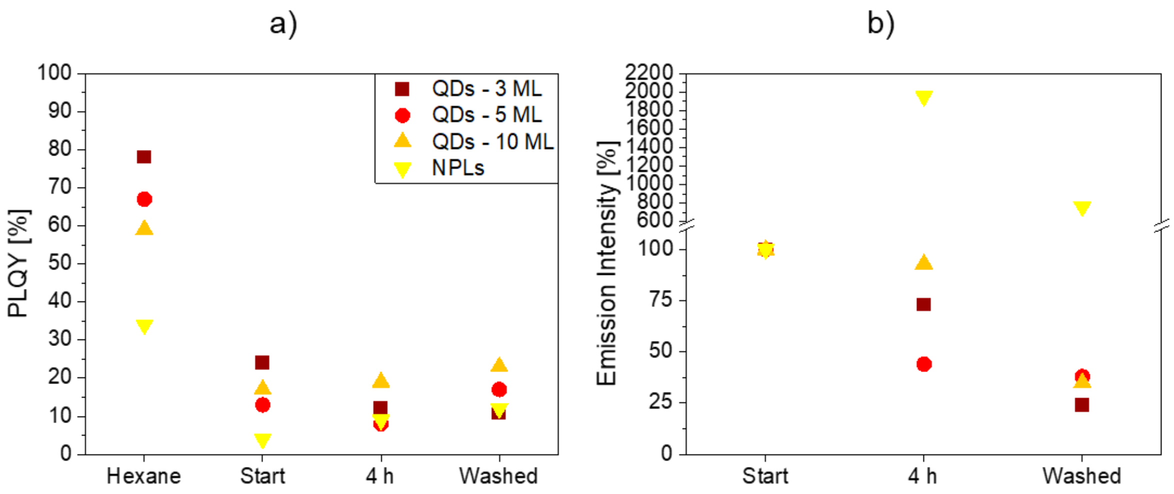

Figure S7: a) PLQY values and b) emission intensity of all NPs that showed sufficient PL properties in hexane and during the polymerization reaction. The emission intensity was defined as 100% at the start of the reaction. All values displayed here, as well as the shift in emission maxima, are also summarized in the SI (see Table S1).

8. STEM images of PSMPs stained with QDs from previous works

In Figure S8, STEM images of different, QD stained PSMPs are displayed. The images highlight the importance of not only NP type, but also the polymerization procedure parameters. The bead displayed in Figure S8 a) was synthesized with different polymerization parameters (e.g., slower stirring speed), and shows the CdSe/CdS QDs located in the bead core region, contrary to the QDs being located in the bead surface region which was observed for the PSMPs in this work. Figure S8 b) shows a COOH-functionalized PSMP stained with CdSe/Cds QDs (10 ML shell thickness) located in the bead surface region, which confirms that the QD location is very similar to plain PSMP stained with the same QDs.

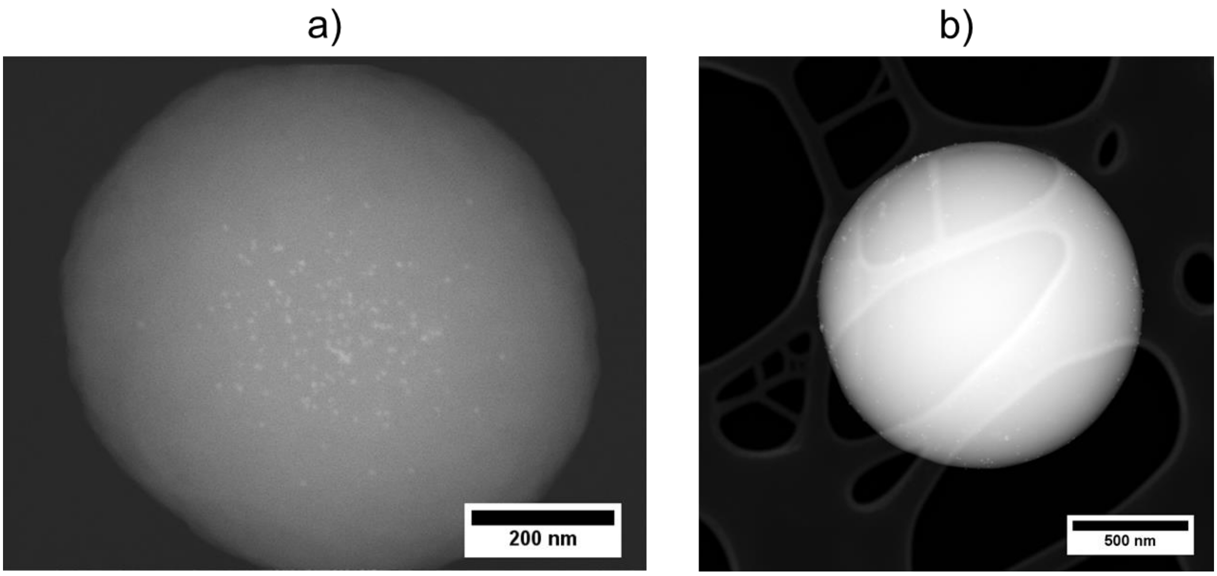

Figure S8: STEM images of QD stained PSMPs, with a) CdSe/CdS QDs (5 ML shell thickness) located in the bead core region, and b) CdSe/CdS QDs (10 ML shell thickness) located in (and even on) the bead surface region. The pictured particles were produced previously by us (see Scholtz *et al.* (2022)[4] for particles in a) and Scholtz *et al.* (2023)[5] for particles in b)).

References

1 Carbone, L. *et al.* Synthesis and micrometer-scale assembly of colloidal CdSe/CdS nanorods prepared by a seeded growth approach. *Nano letters* **7,** 2942-2950, doi:10.1021/nl0717661 (2007).

2 Nightingale, A. M. *et al.* Large-scale synthesis of nanocrystals in a multichannel droplet reactor. *Journal of Materials Chemistry A* **1,** 4067-4076, doi:10.1039/C3TA10458C (2013).

3 Chen, O. *et al.* Compact high-quality CdSe–CdS core–shell nanocrystals with narrow emission linewidths and suppressed blinking. *Nature materials* **12,** 445-451, doi:10.1038/nmat3539 (2013).

4 Scholtz, L. *et al.* Luminescence encoding of polymer microbeads with organic dyes and semiconductor quantum dots during polymerization. *Scientific Reports* **12,** 12061, doi:10.1038/s41598-022-16065-x (2022).

5 Scholtz, L. *et al.* Influence of nanoparticle encapsulation and encoding on the surface chemistry of polymer carrier beads. *Scientific Reports* **13,** 11957, doi:10.1038/s41598-023-38518-7 (2023).

6 Carbone, L. *et al.* Synthesis and Micrometer-Scale Assembly of Colloidal CdSe/CdS Nanorods Prepared by a Seeded Growth Approach. *Nano Letters* **7,** 2942-2950, doi:10.1021/nl0717661 (2007).

7 Nightingale, A. M. *et al.* Large-scale synthesis of nanocrystals in a multichannel droplet reactor. *Journal of Materials Chemistry A* **1,** 4067-4076, doi:10.1039/C3TA10458C (2013).

8 Boercker, J. E. *et al.* Synthesis and Characterization of PbS/ZnS Core/Shell Nanocrystals. *Chem Mater* **30,** 4112-4123, doi:10.1021/acs.chemmater.8b01421 (2018).

9 Chen, O. *et al.* Compact high-quality CdSe–CdS core–shell nanocrystals with narrow emission linewidths and suppressed blinking. *Nature Materials* **12,** 445-451, doi:10.1038/nmat3539 (2013).

10 Tessier, M. D. *et al.* Efficient Exciton Concentrators Built from Colloidal Core/Crown CdSe/CdS Semiconductor Nanoplatelets. *Nano Letters* **14,** 207-213, doi:10.1021/nl403746p (2014).

11 Abécassis, B., Tessier, M. D., Davidson, P. & Dubertret, B. Self-Assembly of CdSe Nanoplatelets into Giant Micrometer-Scale Needles Emitting Polarized Light. *Nano Letters* **14,** 710-715, doi:10.1021/nl4039746 (2014).

12 Miethe, J. F., Schlosser, A., Eckert, J. G., Lübkemann, F. & Bigall, N. C. Electronic transport in CdSe nanoplatelet based polymer fibres. *Journal of Materials Chemistry C* **6,** 10916-10923, doi:10.1039/C8TC03879A (2018).

13 Rossinelli, A. A. *et al.* High-temperature growth of thick-shell CdSe/CdS core/shell nanoplatelets. *Chemical Communications* **53,** 9938-9941, doi:10.1039/C7CC04503D (2017).

14 Meier, M. A. R., Aerts, S. N. H., Staal, B. B. P., Rasa, M. & Schubert, U. S. PEO-b-PCL Block Copolymers: Synthesis, Detailed Characterization, and Selected Micellar Drug Encapsulation Behavior. *Macromolecular Rapid Communications* **26,** 1918-1924, doi:10.1002/marc.200500591 (2005).
